# Selective Binding and Redox-Activity on Parallel G-Quadruplexes by Pegylated Naphthalene Diimide-Copper Complexes

**DOI:** 10.3390/molecules26165025

**Published:** 2021-08-19

**Authors:** Valentina Pirota, Enrico Lunghi, Alessandra Benassi, Emmanuele Crespan, Mauro Freccero, Filippo Doria

**Affiliations:** 1 Department of Chemistry, University of Pavia, Via Taramelli 10, 27100 Pavia, Italy; enrico.lunghi01@universitadipavia.it (E.L.); alessandra.benassi01@universitadipavia.it (A.B.); mauro.freccero@unipv.it (M.F.); 2 “G-Quadruplexes as INnovative ThERApeutiC Targets” (G4-INTERACT), Universal Scientific Education and Research Network (USERN), Via Taramelli 10, 27100 Pavia, Italy; 3 Institute of Molecular Genetics IGM-CNR “Luigi Luca Cavalli-Sforza”, Via Abbiategrasso 207, 27100 Pavia, Italy; crespan@igm.cnr.it

**Keywords:** G-quadruplex, copper-complexes, naphthalene diimide, G-quadruplex-selective ligand, copper redox activity

## Abstract

G-quadruplexes (G4s) are higher-order supramolecular structures, biologically important in the regulation of many key processes. Among all, the recent discoveries relating to RNA-G4s, including their potential involvement as antiviral targets against COVID-19, have triggered the ever-increasing need to develop selective molecules able to interact with parallel G4s. Naphthalene diimides (NDIs) are widely exploited as G4 ligands, being able to induce and strongly stabilize these structures. Sometimes, a reversible NDI-G4 interaction is also associated with an irreversible one, due to the cleavage and/or modification of G4s by functional-NDIs. This is the case of **NDI-Cu-DETA**, a copper(II) complex able to cleave G4s in the closest proximity to the target binding site. Herein, we present two original Cu(II)-NDI complexes, inspired by **NDI-Cu-DETA**, differently functionalized with 2-(2-aminoethoxy)ethanol side-chains, to selectively drive redox-catalyzed activity towards parallel G4s. The selective interaction toward parallel G4 topology, controlled by the presence of 2-(2-aminoethoxy)ethanol side chains, was already firmly demonstrated by us using core-extended NDIs. In the present study, the presence of protonable moieties and the copper(II) cavity, increases the binding affinity and specificity of these two NDIs for a telomeric RNA-G4. Once defined the copper coordination relationship and binding constants by competition titrations, ability in G4 stabilization, and ROS-induced cleavage were analyzed. The propensity in the stabilization of parallel topology was highlighted for both of the new compounds **HP2Cu** and **PE2Cu**. The results obtained are particularly promising, paving the way for the development of new selective functional ligands for binding and destructuring parallel G4s.

## 1. Introduction

Nucleic acid (NA) sequences rich in guanine-basis (Gs) can fold into four-stranded higher-order supramolecular structures known as G-quadruplexes (G4s) [1,2].

Over the past decades, G4s have gained increasing attention in medicinal chemistry being mainly clustered in top genomic regions, as telomeres, gene promoters, open reading frames, and untranslated regions, and not randomly distributed across the cell genome [3]. Indeed, they are recognized as key NA secondary structures involved in the regulation of different biological processes, whose targeting can be exploited as a revolutionary therapeutic approach to tackle a growing list of different pathologies, such as cancer [4], neurodegenerative diseases [5], viral infections [6], not least COVID-19 [7].

In this context, a rapidly growing interest in the targeting of RNA-G4s has recently emerged. As examples, 5′-UTR mRNA-G4s have been recognized as regulators of gene translational processes [8] and multimeric mRNA-G4s are proven sequestering RNA-binding proteins impairing their functions [9]. Moreover, telomeric repeat-containing RNAs folded into G4s (TERRA) are involved in numerous processes, including the regulation of telomerase functioning [10], as well as the regulation of viral activity which can be affected by RNA-G4s folding [11].

Therefore, the discovery of novel small molecules, able to bind selectively with RNA-G4 structures, has become extremely urgent and needed.

Among all, metal complexes have captivated notable attention, not only for their optimal binding properties and low cytotoxicity but also for the opportunity to use metal catalysis to engineer functional ligands able to oxidase and/or cleave G4s [12]. In this context, some of us have recently developed a naphthalene diimide (NDI)-based G4-ligand bearing a copper complex (**NDI-Cu-DETA**) that, in oxidative conditions, generates reactive oxygen species (ROS) responsible for the H-1′ and H-4′ abstraction of deoxyribose moiety with consequent cleavage of the phosphate backbone [13]. The associated DNA cleavage occurs very close to the G4-ligand binding site, due to the short lifetime of ROS, resulting in a highly specific cleavage.

Starting from these promising results, here we decided to develop functional ligands, based on the Cu-DETA copper complex, able to selectively interact with RNA-G4s.

**Figure 1:**
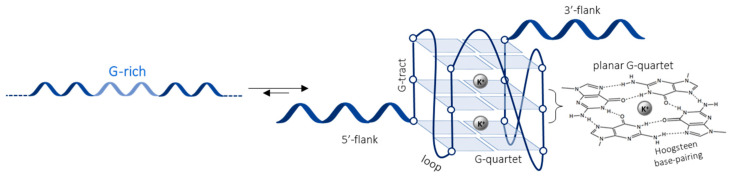
Schematic representation of G-quadruplex structure.

The building blocks of the G4s are the planar guanine quartets, the so-called G-quartets or G-tetrads, which stack on top of each other and are stabilized by metal cations, among all potassium (K^+^) [1]. They are constituted by four guanines, that interact via Hoogsteen-type hydrogen bonds base-pairing (Figure 1), exposing planar aromatic surfaces which represent a suitable binding site. Accordingly, most G4 ligands consist of a flat-shaped aromatic system able to interact on the top of the terminal G-quartets by π-stacking (end-stacking), as the above-mentioned NDIs [14,15]. This specific geometric feature ensures strong stabilization and high selectivity towards G4s compared to other NA structures.

To maximize ligands’ specificity for a unique G4 target, differentiating structural elements, such as loops, flanking regions, and G4-topologies (Figure 1), must be identified and exploited.

In this context, an effective strategy to selectively target RNA-G4s may take advantage of their common parallel topology. The presence of 2′-hydroxyl groups on ribose sugars exerts orientation of the G bases about the glycosidic bond to an anti-conformation, imparting C3′-endo puckering and limiting the G4 topology to a parallel one, which is thermodynamically more stable [16].

Recently, we have demonstrated that the introduction of 2-(2-aminoethoxy)ethanol side-chains on core-extended NDIs not only provides a remarkable selectivity for G4s over other NA structures but promotes specific stabilization of parallel G4s [17]. Nevertheless, in this case, the complete absence of electrostatic interactions with the target, due to the lack of charged amino groups, decreases the binding affinity of the ligand.

**Scheme 1:**
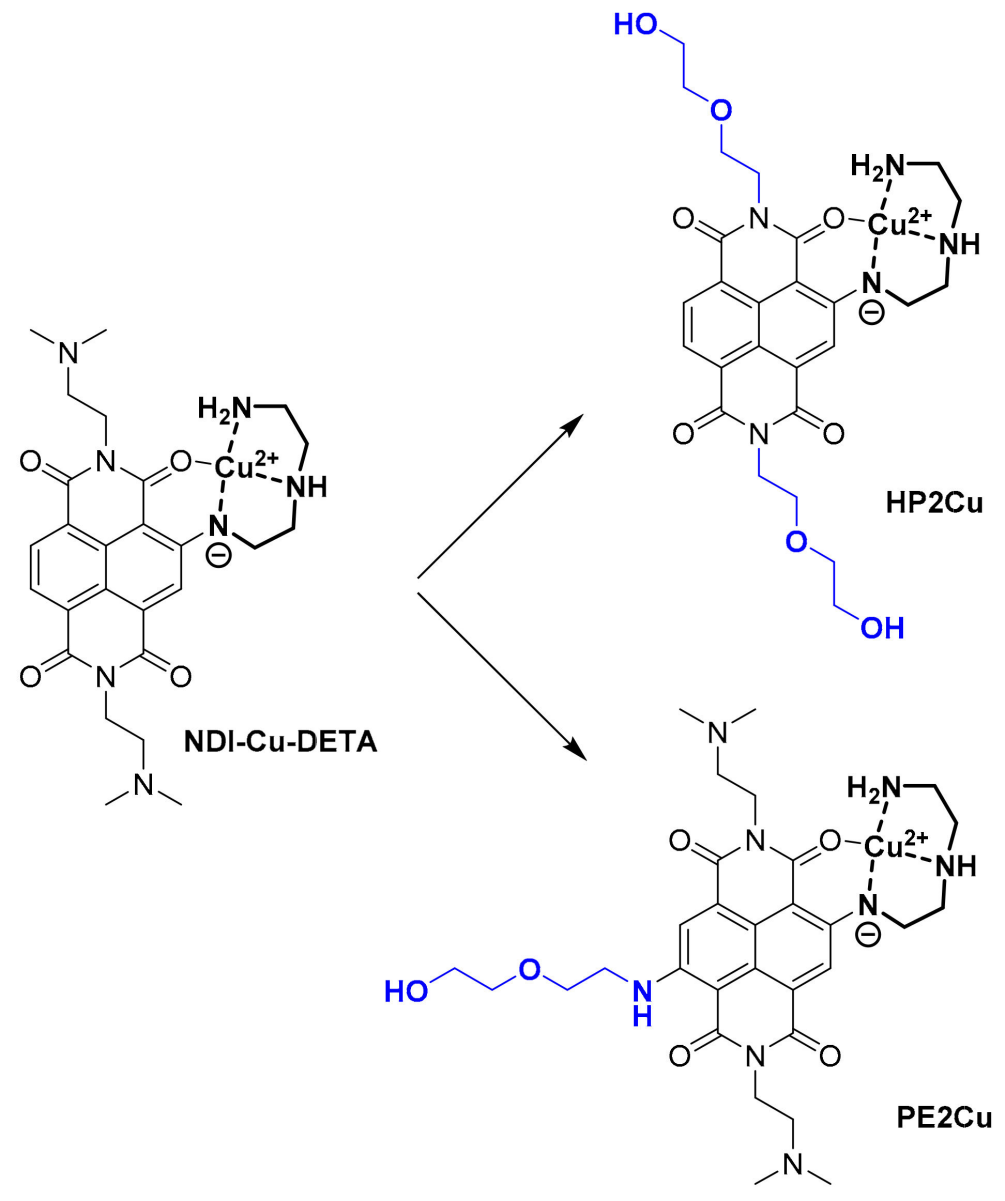
Chemical structures of previously published **NDI-Cu-DETA** [13] and of the new Cu-DETA-substituted NDIs **HP2Cu** and **PE2Cu**.

Therefore, starting from the chemical structures of the previously published **NDI-Cu-DETA** [13], we designed and synthesized two novel Cu-DETA-substituted NDIs, **HP2Cu** and **PE2Cu** (Scheme 1), introducing 2-(2-aminoethoxy)ethanol pendants at both the imide positions, or as a core-side chain, respectively.

Spectroscopic features and copper coordination of the new complexes are here analyzed as well as the ROS species generation by copper redox catalysis. We investigated the specific interaction of **HP2Cu** and **PE2Cu** towards two telomeric G4s: htel22, a hybrid DNA-G4, and TERRA, a parallel RNA-G4 (for NA sequence, please refer to Table S1, Supplementary Material). We decided to use these two G4s as a proof of concept, being constituted by the same nucleic acid sequence, with the exception of Uracils in place of Thymines in the case of TERRA RNA, and for which the two G4 topologies (hybrid vs. parallel) only depend on the difference between the sugars. Once we analyzed the G4 stabilization induced by new Cu-complexes **HP2Cu** and **PE2Cu**, their abilities in the NA cleavage under oxidative conditions were explored.

Overall, this work aims to obtain novel functional G4-ligands, based on copper-complexes, able to selectively interact with parallel G4 structures, among which the most numerous and the ones with a greater folding propensity are RNA-G4s.

## 2. Results and Discussion

### 2.1. Synthesis of the New DETA-Substituted NDIs **HP2** and **PE2**

The synthesis of these two new Cu-complexes derived from a combination of previously reported strategies.

**Scheme 2:**
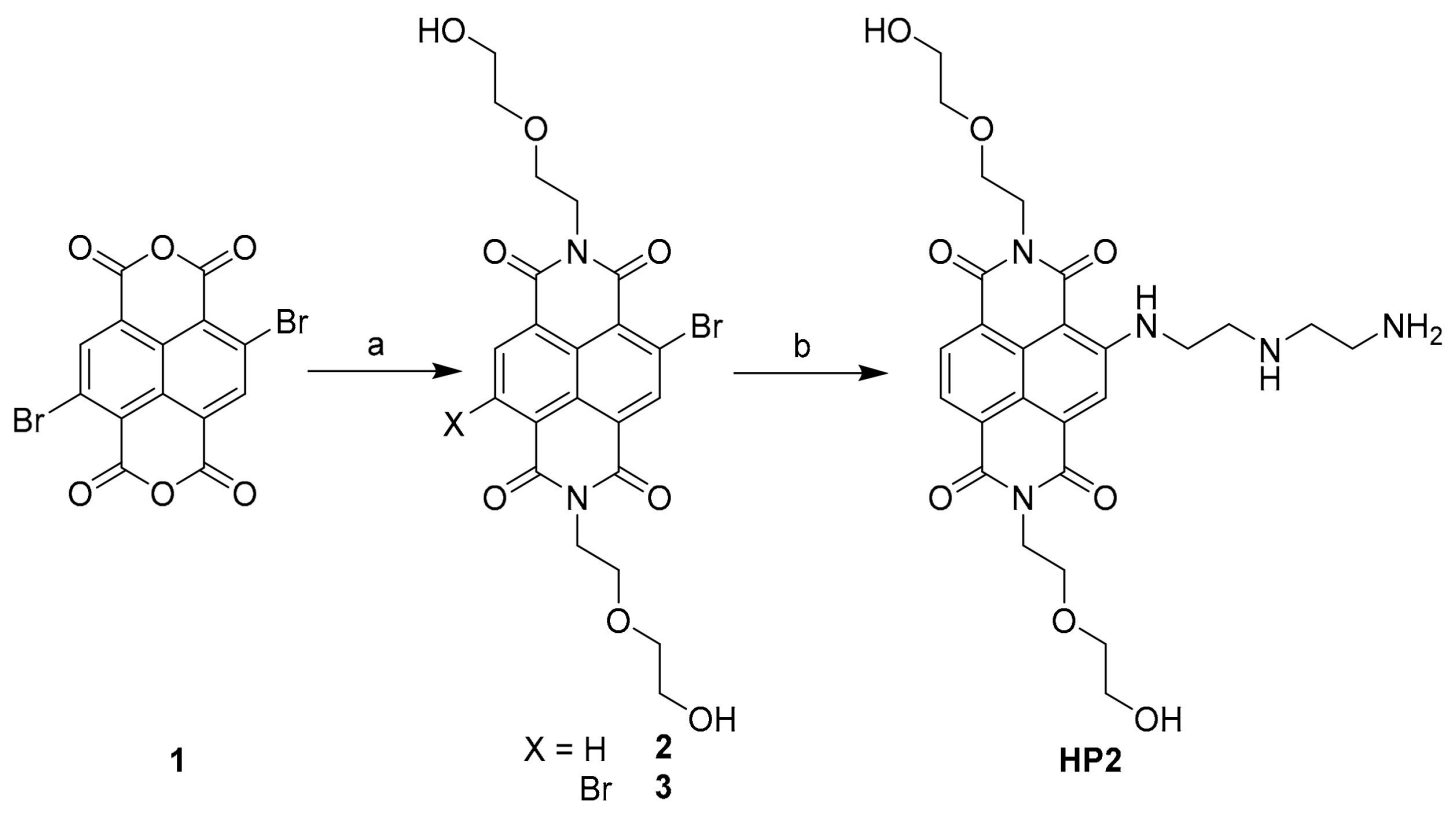
Synthesis of **HP2**: (a) 2-(2-aminoethoxy)ethanol (2 eq.) in 55:45 EtOH:CH_3_COOH, 5 min, 120 °C, 210 psi, mw assisted; (b) diethylentriamine (8 eq.) in DMA, 45 °C, 3 h.

In brief, the synthetic protocol to obtain **HP2** started with the imidation of **1**, obtained as previously described [18], with 2 equivalents of 2-(2-aminoethoxy)ethanol in a 55:45 mixture of EtOH:CH_3_COOH at 120 °C (210 psi), assisting the reaction by microwaves (Scheme 2, step a). This step was modified from a previously published procedure, to reduce reaction time and increase the yield [17]. As previously, partial dehalogenation of the naphthalene core occurred, leading compounds **2** and **3** as a blend, which was used without purification. The reaction course was monitored by analytical HPLC, comparing the results with those gained with the previous protocol [17]. To introduce the copper-binding unit, a nucleophilic aromatic substitution (S_N_Ar) was performed with an excess of commercial diethylentriamine (DETA) in dimethylacetamide (DMA), at 45 °C (Scheme 2, step b) [13]. After 3 h, the crude was purified by preparative HPLC, isolating **HP2** as a red solid.

**Scheme 3:**
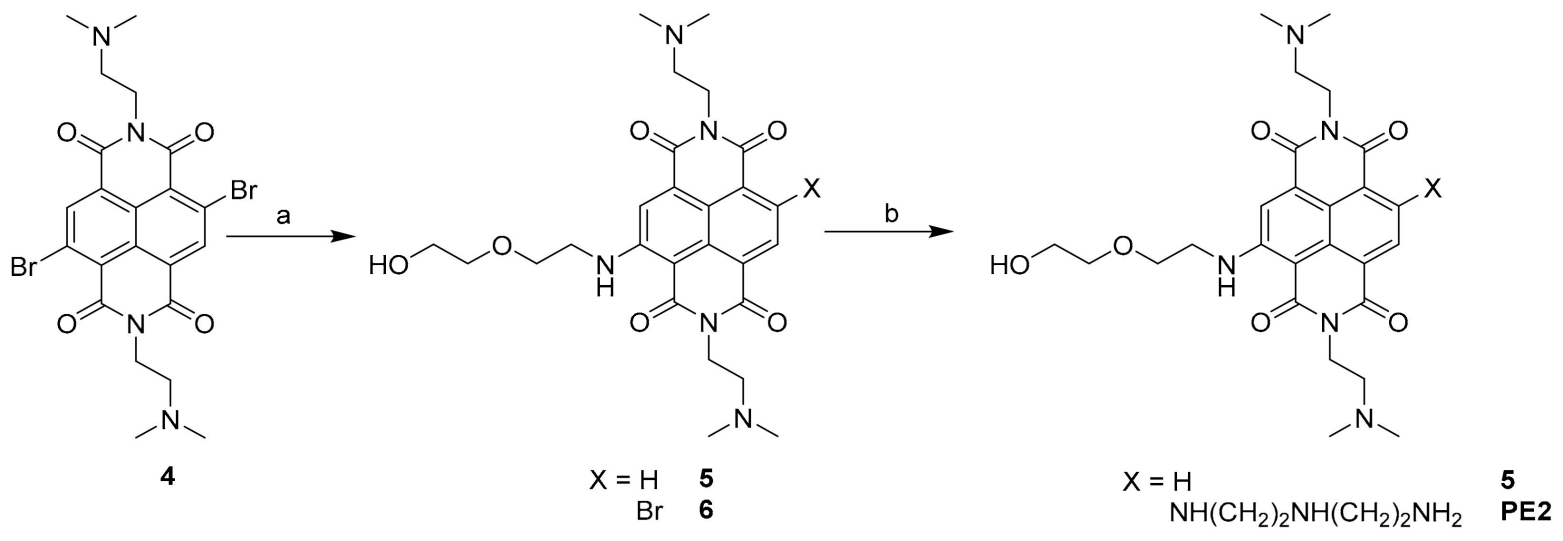
Synthesis of **PE2**: (a) 2-(2-aminoethoxy)ethanol (2.5 eq.) in CH_3_CN, 4 h, 80 °C; (b) diethylentriamine (8 eq.) in DMF, 45 °C, o.n.

To synthesize **PE2**, a thermal S_N_Ar on the naphthalene core of compound **4**, synthesized as previously reported [19], was performed. Compound **4** was dissolved in acetonitrile, then 2.5 equivalents of 2-(2-aminoethoxy)ethanol were added and the solution was refluxed for 4 h, following the reaction course by UPLC-ESI-MS (Scheme 3, step a). The solvent was removed under vacuum and the resulting red crude was resuspended in hydrogen carbonate saturated water and extracted with dichloromethane, leading to a mixture of compounds **5** and **6**. This latter was dissolved in dimethylformamide (DMF), and an excess of commercial DETA was added to perform a second S_N_Ar on reactive compound **6**. The solution was stirred at 45 °C overnight, then the solvent was removed under reduced pressure and the crude purified by preparative HPLC to isolate compound **5** and the desired **PE2** as a violet solid (Scheme 3, step b).

### 2.2. Copper Coordination to **HP2** and **PE2** and Complexes Synthesis

To evaluate the copper-complexes formation, spectrophotometric titrations were recorded in 0.1 M HEPES buffer, pH 7.4, using a cuvette of 10 cm optical path. Increasing amounts of a millimolar stock solution of Cu(OTf)_2_ (from 0 to 3 equivalents) were added to 5 µM of NDI-ligands.

**Figure 2:**
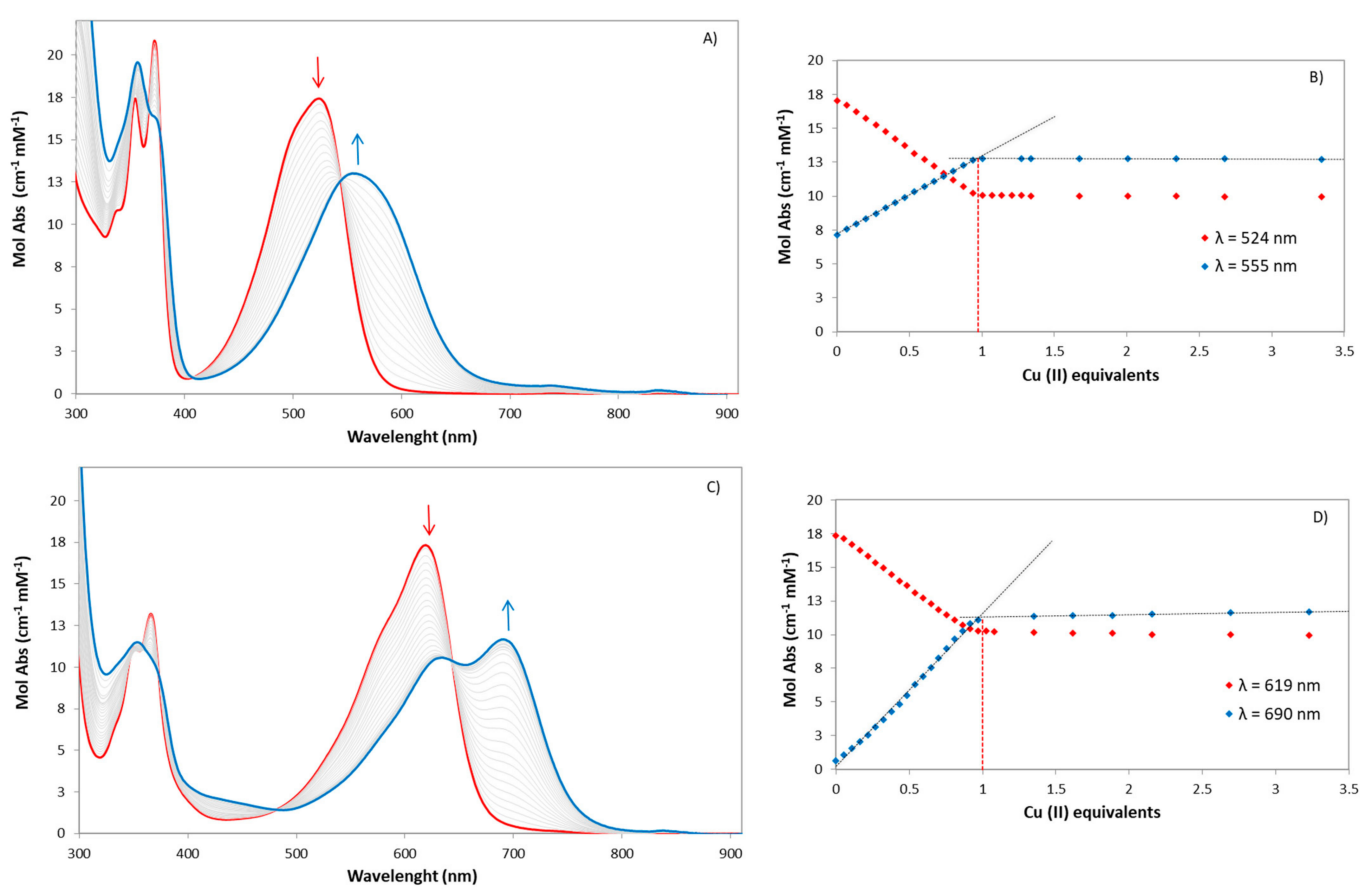
Spectrophotometric titration spectra of 5 µM (**A**) **HP2** and (**C**) **PE2** with increasing amounts of Cu(II) in 0.1 M HEPES buffer, pH 7.4, 25 °C, in a cell of 10 cm optical length. Molar absorption profiles at (**B**) 524 (red diamonds) and 555 (blue diamonds) nm for **HP2Cu** complexation and at (**D**) 619 (red diamonds) and 690 (blue diamonds) nm for **PE2Cu** complexation.

Absorption spectra features of NDIs are strictly related to the electronic nature of the substituent on the naphthalene core. In particular, **HP2** shows a charge-transfer (CT) band at 524 nm, due to the electronic contribution of DETA substituent on NDI core, that decrease in function of copper addition from 0 to 1 equivalent. The formation of the **HP2Cu** complex redshifts the CT band of 31 nm (λ_max_ = 555 nm), and, accordingly, the color of the solution turned from orange to violet (Figure 2A). By plotting the molecular absorptivity at 524 nm and 555 nm as a function of copper equivalents and interpolating the experimental points by two straight lines, it was possible to identify the value 0.992 ± 0.003 as the point of intersection (Figure 2B). This 1:1 binding mode suggests an impressive binding constant for Cu(II) uptake, as expected from previous results [13].

The presence of a second substituent on the naphthalene core increases the electron density on NDI, inducing a more pronounced bathochromic shift of the CT band of **PE2** up to 619 nm. Accordingly, the addition of Cu(II) induced a further redshift, generating a double CT band for **PE2Cu** complex, with the second maximum at 690 nm (Figure 2C), resulting in a color change from violet to blue. As in the previous case, complex formation occurs with a 1:1 binding mode, as highlighted by the insertion of the two interpolating straight lines at 1.001 ± 0.005 (Figure 2D).

**Scheme 4:**
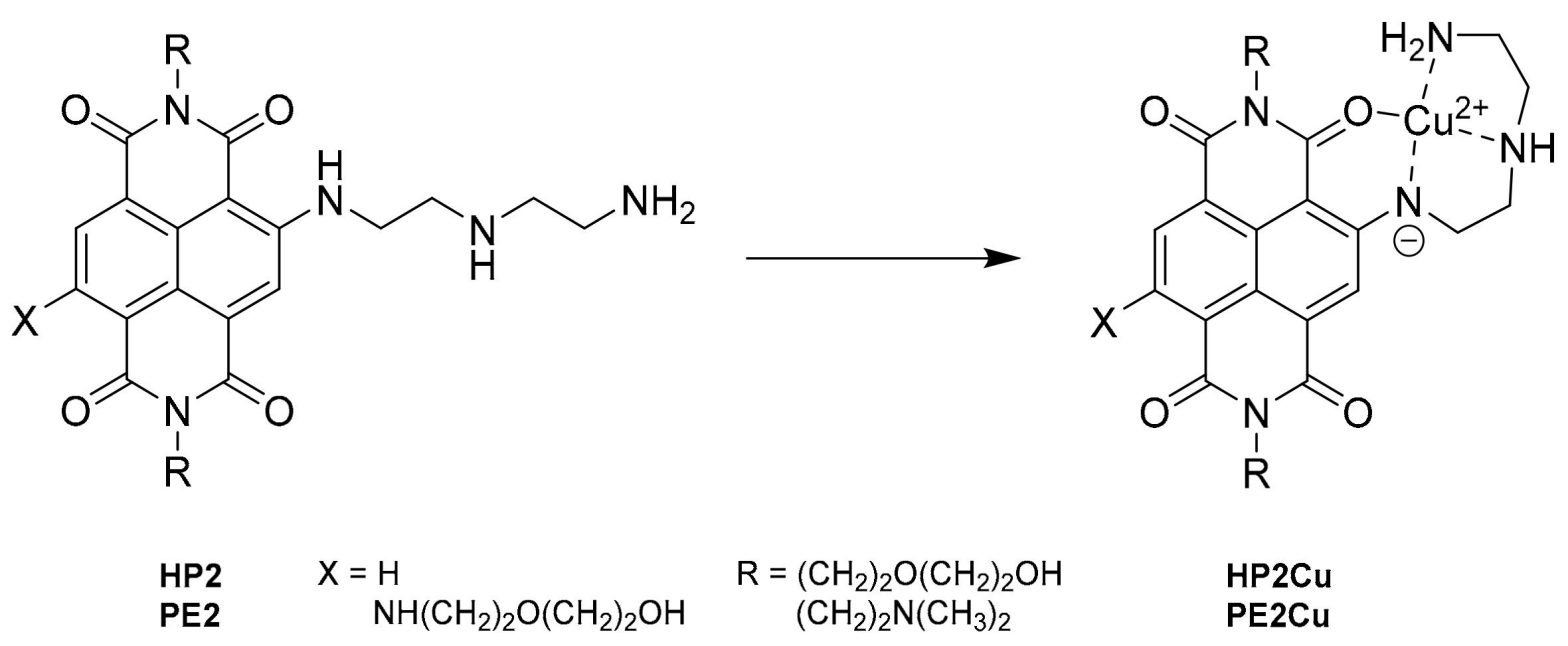
Synthesis of copper-complexes. NDI ligands (**HP2** or **PE2**), Cu(ClO_4_)_2_·6H_2_O (1 eq.) in distilled H_2_O, KPF_6_, pH 7.0.

After having verified the high coordination efficiency of the Cu(II) ion by our two NDI-ligands, we proceeded with Cu(II)-complexes synthesis using the same protocol previously exploited for **NDI-Cu-DETA** [13]. The NDI ligand (**HP2** or **PE2**) was dissolved in distilled water adding a stoichiometric amount of Cu(ClO_4_)_2_ hexahydrate salt. The pH was moved to 7.0 and an excess of KPF_6_ was added to the solution, forcing **HP2Cu** (as violet solid) or **PE2Cu** (as blue solid) complex precipitation (Scheme 4). The water solution was removed by hypercentrifugation and the solid was dried by treating it with cooled diethyl ether.

### 2.3. Determination of the Binding Constants

Considering the high propensity of these two new NDI-ligands in copper coordination, competition titration experiments were performed to assess the binding constants of the Cu(II)-complexes.

Based on previous results [13], tris(2-aminoethyl)amine (TREN) was selected as a competing ligand, because of its ability to form a stable 1:1 complex with Cu(II) in water at physiological pH with a binding constant of 18.8 Log units [20].

**Figure 3:**
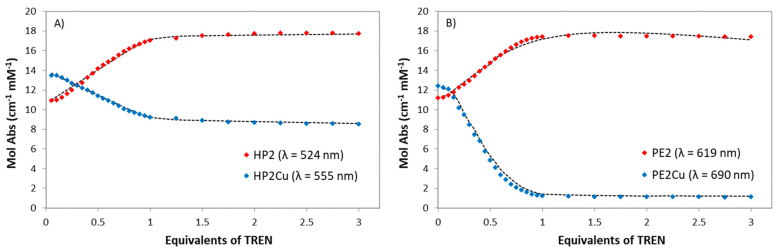
Spectrophotometric competitive titration of Cu(II)-NDI complexes with TREN. Here we reported the distribution diagrams of the species for the titrations of (**A**) **HP2Cu** and (**B**) **PE2Cu** with increasing equivalents of TREN in 0.1M HEPES buffer, pH 7.4, 25 °C.

**Scheme 5:**
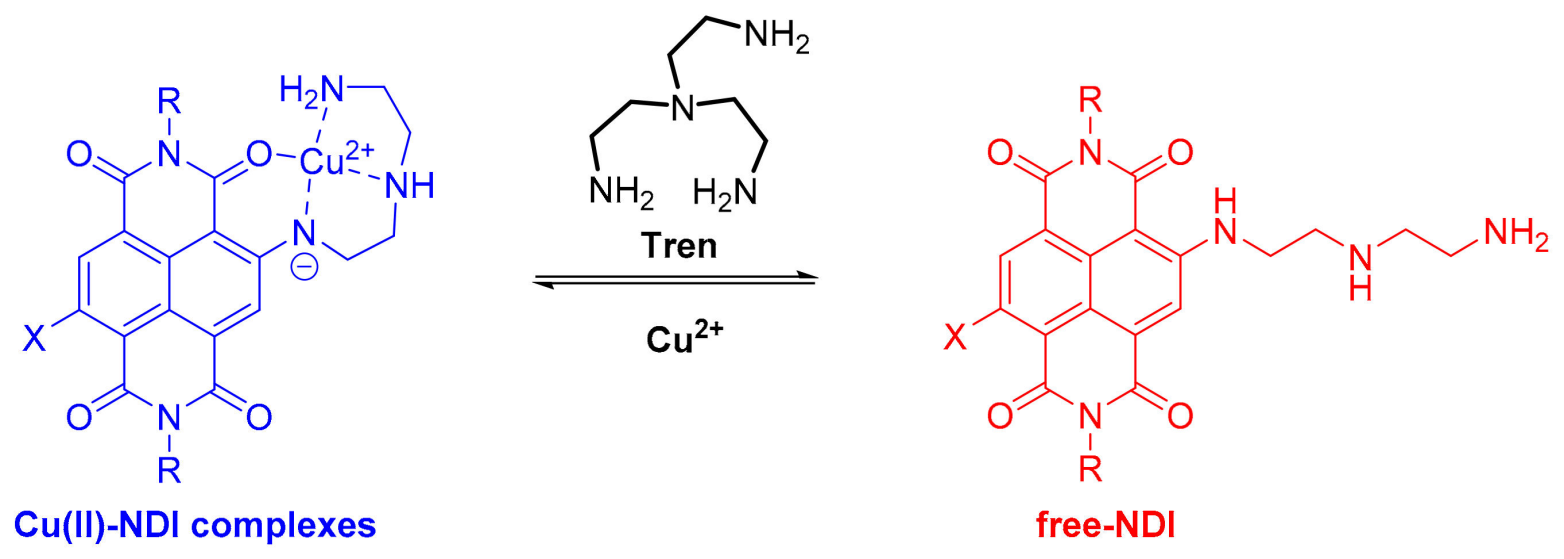
Schematic representation of the competitive titration of Cu(II)-NDI complexes with TREN to obtain free-NDI ligands. The addition of Cu(II) to free-NDI restores the Cu(II)-NDI complexes.

**HP2Cu** or **PE2Cu** were dissolved in 0.1 M HEPES buffer at pH 7.4, obtaining a 50 µM solution which was titrated with increasing amounts of TREN (Scheme 5). As expected, TREN competed for Cu(II)-binding, displacing Cu(II) from NDI ligand, as it is easily deductible from the decrease of the CT bands intensity, associated to Cu(II)-NDI complexes (555 nm for **HP2Cu** and 690 nm for **PE2Cu**). Absorption spectra of the free-NDI ligands were completely restored after the addition of 1 equivalent of TREN (Figure S1 and Figure 3).

Binding constants were coherently estimated as 16.30 ± 0.08 Log units for **HPCu2** and 17.95 ± 0.02 Log units for **PE2Cu**. Both these results are in agreement with data obtained for **NDI-Cu-DETA** (Logβ = 17.3).

One order of magnitude of difference between **HP2Cu** and **NDI-Cu-DETA** Logβ values may depend on the NDI imide pendants. Dimethyl-substituted nitrogen of the *N*,*N*-dimethylethylenediamine chain in **NDI-Cu-DETA** could offer a stronger fifth Cu(II)-coordination position than the oxygen of the 2-(2-aminoethoxy)ethanol present in **HP2Cu**, in perfect accordance with Pearson’s acid-base concept and the Irving–Williams series.

On the contrary, the presence of a tetra-substitution on **PE2Cu**, increases the electron density on NDI, making the imide oxygen more available to Cu(II)-coordination.

### 2.4. Analysis of the Effect of **HP2Cu** and **PE2Cu** on Telomeric G-Quadruplexes

Impressive binding constant at physiological conditions, water solubility, and intriguing optical properties prompted us to study **HP2Cu** and **PE2Cu** as G4 ligands. Two sequences, TERRA and hTel22, were chosen since they are essentially composed of the same base sequence (22 nucleotides). In spite of this, their different nature, RNA versus DNA, respectively, together with the presence of Uracils instead of Thymines, offer the opportunity to compare a parallel G4 topology (TERRA) to a hybrid one (hTel22) [21,22].

Circular dichroism (CD) spectra, performed in 10 mM lithium cacodylate buffer at pH 7.4 with different potassium concentrations (see Table 1), confirmed the parallel topology of TERRA, featured by a positive maximum at 264 nm and a negative minimum around 240 nm, while hTel22 folded into a hybrid G4, characterized by a positive maximum at 290 nm with a shoulder at 270 nm [23].

To explore the well-recognized G4-ligand ability of the NDI core in relationship with the contribution due to the presence of the new side chains on G4 topology selectivity, CD-melting experiments (from 20 °C to 90 °C) were performed. The melting temperature values (T_m_) of oligonucleotides alone were compared with those measured in the presence of 2 equivalents of **HP2Cu** or **PE2Cu**, left to equilibrate with the G4s for 16 h at 20 °C before recording the CD melting assay. To appreciate the added value of the Peg-like chain introduced, T_m_ stabilization results induced by the new ligands were compared with those obtained in the presence of 2 equivalents of the parent **NDI-Cu-DETA** [13].

As highlighted by the data listed in Table 1, remarkable stabilization of TERRA-G4 has been observed for both **HP2Cu** and **PE2Cu**, with better results obtained for **PE2Cu**, whose presence shifts the value of T_m_ beyond the measured temperature range (>90 °C) at 100, 10, or 1 mM potassium concentrations, and stabilized the RNA-G4 target of 24.2 °C in the presence of a very small amount of KCl (0.1 mM). The stabilization effects of the parent **NDI-Cu-DETA** on the RNA target are substantially comparable to those obtained with **HP2Cu** at high and medium potassium concentrations (100 and 10 mM). As the contribution of potassium in stabilizing the target decreases (1 and 0.1 mM), electrostatic interactions due to *N*,*N*-dimethylethylenediamine imide chains of **NDI-Cu-DETA**, protonated in physiological conditions, are more efficient in stabilizing TERRA, compared to hydrogen bond interactions of **HP2Cu**. These results confirmed that the introduction of at least one 2-(2-aminoethoxy)ethanol chain greatly favors the interaction with parallel G4 topology, as previously extensively studied with core-extended NDIs [17]. In addition, the remarkable differences between **HP2Cu** and **PE2Cu**, and the results obtained with **NDI-Cu-DETA**, suggest that the presence of protonable side chains at physiological conditions, as *N*,*N*-dimethylethylenediamine in **PE2Cu**, further reinforces the interaction with the G4 structure, as expected.

**Figure 4:**
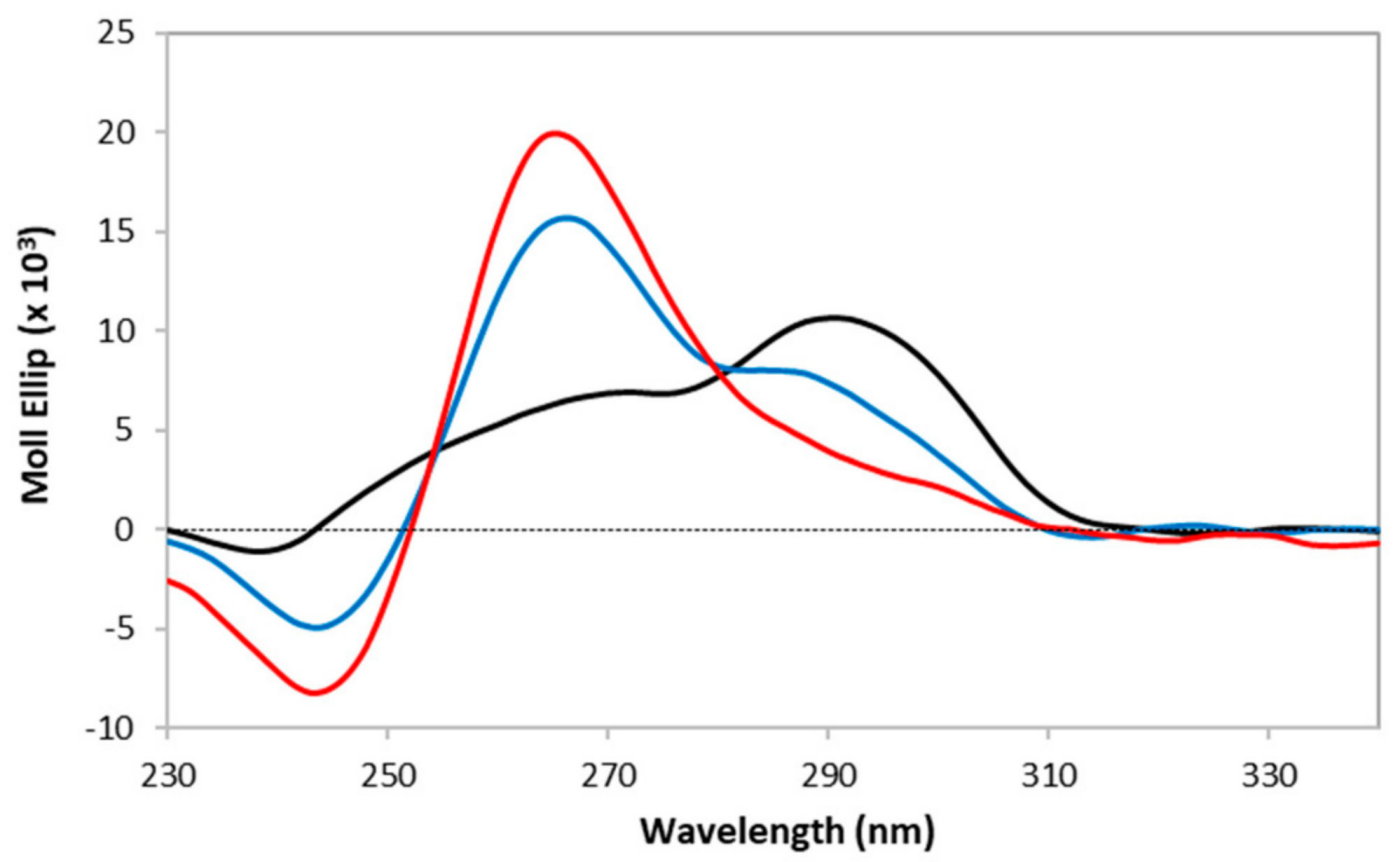
CD spectra of 5 µM hTel22 alone (black line), after the addition of 2 eq. of **PE2Cu**, left to equilibrate at room temperature for 16 h (blue line), and after the hTel22 G4 folding in the presence of 2 eq. of **PE2Cu** (red line) in 10 mM lithium cacodylate buffer at pH 7.4 with 100 mM KCl.

Of particular interest is the analysis of the effect that these ligands have on the hybrid topology of hTel22. Unlike **NDI-Cu-DETA**, neither of the two new Cu(II)-NDI ligands showed significant stabilization of hTel22 original topology, as is highlighted by Tm values identified at 290 nm. Remarkably, **PE2Cu** exhibited an unexpected destabilization of the target, decreasing the melting temperature by almost 17 °C and 13 °C at 100 mM and 10 mM potassium concentrations, respectively. These results are in accordance with the significant modification of the original dichroic footprint of hTel22, also at high potassium concentrations, resulting from the addition of 2 equivalents of **PE2Cu** (blue line in Figure 4). Following the melting temperature of hTel22 at 264 nm, it was possible to evince a strong G4 stabilization by **PE2Cu** higher than 11.6 °C in presence of 100 mM KCl, and of 23 °C with 10 mM KCl (Table 1). Much more fascinating was the fact that the folding process in the presence of **PE2Cu** is not reversible at all. The dichroic signal recorded after the rebalancing of the 1:2 hTel22:**PE2Cu** mixture from 90 °C to 20 °C, over 30 min, showed a dominant parallel topology, underlining a drastic reorganization of the nucleic bases forced by **PE2Cu** (red line in Figure 4). Therefore, **PE2Cu** is clearly able to induce a complete shift in the hTel22 topology, from hybrid (black line in Figure 4) to parallel (red line in Figure 4). Moreover, the T_m_ value of this new G4-**PE2Cu** complex was greater than 90 °C (Table S2), pointing out the high affinity of this complex for the modified topology of the target.

Although the hTel22 stabilization induced by **NDI-Cu-DETA** at 264 nm is evenly considerable (Table 1), the effect on the G4 topology is not quite comparable. The addition of 2 equivalents of **NDI-Cu-DETA** increased the molecular ellipticity of the hTel22 dichroic signal, leaving the topology almost unchanged (Figure S2, blue line). A partial topology reorganization is appreciable only if the folding hTel22 was performed in the presence of **NDI-Cu-DETA** (Figure S2, red line), nevertheless, the CD-signal obtained corresponds to a mixture of topologies in which parallel one is not predominant.

Conversely, no topology changes were measured in the presence of **HP2Cu** (Table S2 and Figure S3). This could suggest a lower affinity towards a hybrid DNA-G4 topology, strongly stabilized by the presence of potassium (100 mM). As in the previous case, the decrease in potassium concentration, and therefore the presence of a G4 structure less stabilized by the metal cation, allows **HP2Cu** to increase its stabilization capacity (Table 1).

Comparing these results, it can be assumed that it is not only the presence of Peg-like chains but also their relative disposition on the ligand that increases the affinity towards G4 parallel topologies.

### 2.5. Evaluation of ROS-Mediated NA Cleavage

To validate **HP2Cu** and **PE2Cu** functional targeting due to peculiar oxidative established chemistry on DNA, CD spectra variation of TERRA parallel G4 induced by sodium ascorbate/Cu(II)-NDI/H_2_O_2_ system were followed. The oxidative effect of free Cu(II) ion in presence of sodium ascorbate (1 mM) and H_2_O_2_ (1 mM) on TERRA was compared with those obtained in the presence of the two Cu(II)-NDI ligands.

**Figure 5:**
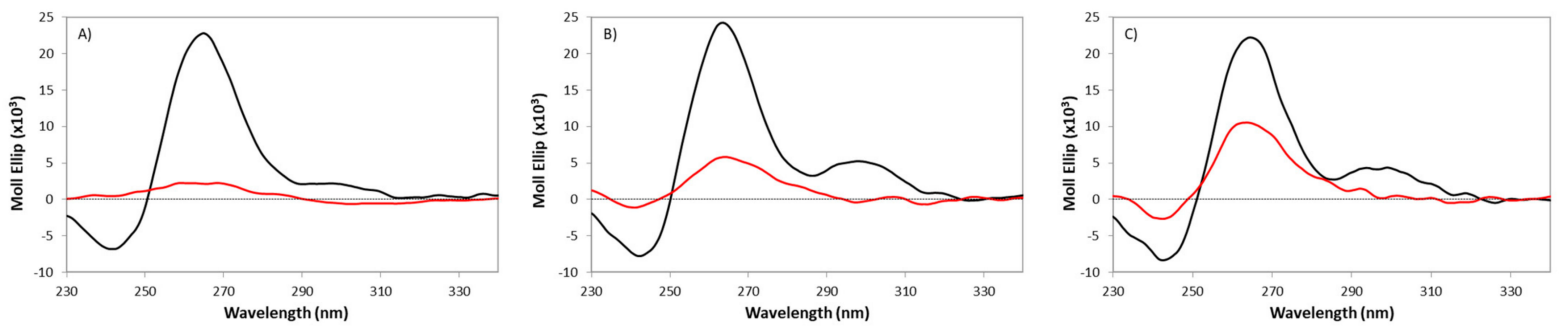
CD spectra of TERRA (5 µM) in presence of 1 eq. of (**A**) Cu(ClO_4_)_2_, (**B**) **HP2Cu** or (**C**) **PE2Cu** in 10 mM lithium cacodylate buffer at pH 7.4 with 100 mM KCl (black lines). In all the solutions, sodium ascorbate (1 mM) and H_2_O_2_ (1 mM) were added, and CD spectra were recorded after 2 h reaction (red lines).

Free Cu(II), in the presence of sodium ascorbate and H_2_O_2_, entirely erases the characteristic dichroic signal of TERRA-G4, transforming it into something unstructured, as can be seen from the CD spectrum in Figure 5A (red line).

Preliminary studies on oxidative reactivity of 1:1 **HP2Cu** or **PE2Cu** (5 µM) on TERRA (5 µM) suggested a catalyzed oxidative pathway that localized the damage on specific bases of the NA structure. As a matter of fact, the CD spectra of TERRA were partially maintained (Figure 5B,C). Moreover, the more intense dichroic signal of **PE2Cu** suggests a higher affinity for the target and less freedom to randomly cleavage the G4 structure.

A very preliminary evaluation of the sequence-specific cleavage of **HP2Cu** and **PE2Cu** in presence of sodium ascorbate (1 mM) and H_2_O_2_ (1 mM), was obtained by denaturing polyacrylamide gel electrophoresis (PAGE) assay performed on 5′-FAM-TERRA oligonucleotide (Figure S4). Time-dependent cleavage at 37 °C was evaluated after 16 h equilibration of 2 equivalents of Cu(II)-NDI ligands with 5 µM oligonucleotide at 20 °C in 10 mM TRIS-HCl buffer (pH 7.4) with 100 mM KCl. Despite the RNA target being greatly subjected to hydrolytic degradation (Line 13 in Figure S4), as expected, it is possible to identify selective cleavage sites due to the oxidative pathway induced by both the two Cu(II)-NDI ligands. In the case of **PE2Cu**, the principal cleaving site is independent of the reaction time (Line 8–12 in Figure S4), emphasizing the power of this ligand on the target.

### 2.6. Determination of ROS Involved

Reactive oxygen species (ROS) are highly reactive metabolites of oxygen with a very short lifetime (from ns to s) in the biological system [24]. Among all, the most common free radicals obtained as by-products of cellular aerobic metabolism are hydroxyl (OH˙) and superoxide (O_2_ˉ˙) [25]. Two different experiments were performed to identify these specific ROS species potentially involved in NA cleavage.

Hydroxyl radical is a short-lived species that reacts with most organic compounds at nearly diffusion-controlled rates [26]. In this case, Cu(I) can catalyze the decomposition of hydrogen peroxide via a Fenton-like reaction (Reaction 1), which leads to the generation of hydroxyl radicals.

(1) $$Cu^{+}+\;H_{2}O_{2}\;⇌\;Cu^{2+}+\;OH^{-}+OH^{.}\;\;\;\;\;\;\;\;\;\;$$

**Figure 6:**
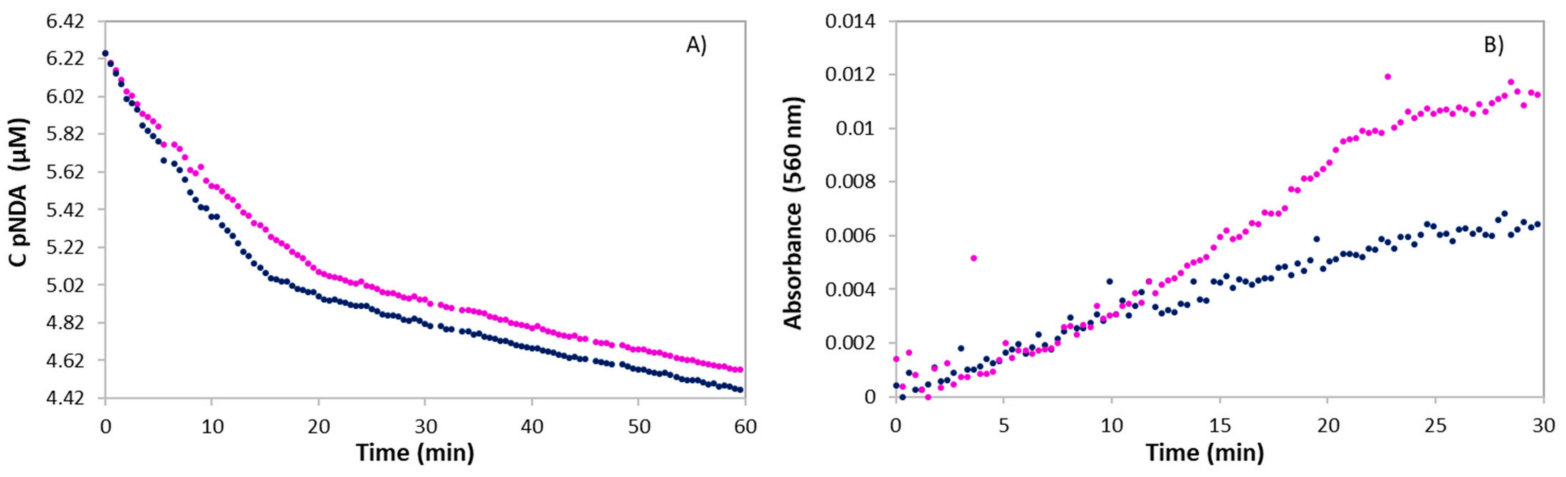
(**A**) Hydroxyl radical-mediated decreasing of *p*-NDA absorbance at 440 nm. Reaction mixtures contained *p*-NDA (6.25 µM) in 10 mM lithium cacodylate buffer (pH 7.4) and the reactions were initiated by the additions of H_2_O_2_ (1 mM), ascorbate (1 mM), and 5 µM **HP2Cu** (purple dots) or 5 µM **PE2Cu** (blue dots). The reaction has been monitored for 60 min at 37 °C. (**B**) Increasing of absorbance at 560 nm, associated with the reduction of NBT (62.5 µM) to formazan, monitored in presence of H_2_O_2_ (1 mM) and ascorbate (1 mM), upon the addition of 5 µM **HP2Cu** (purple dots) or 5 µM **PE2Cu** (blue dots) in KCl (100 mM) and lithium cacodylate buffer (10 mM, pH 7.2), at 37 °C. The reaction has been monitored for 30 min at 37 °C.

Therefore, to confirm the involvement of hydroxyl radical in the G4 cleavage and damage, the bleaching of *p*-nitroso-*N*,*N*-dimethylaniline (*p*-NDA) followed [27,28]. The yellow chromophore-NO group of *p*-NDA was bleached to a colorless -NO_2_ group specifically by ˙OH, with an estimated second-order rate constant of around 10^10^ M^−1^s^−1^ [27]. The bleaching of *p*-NDA was monitored spectrophotometrically at 440 nm (ε _440nm_ = 34,200 M^−1^cm^−1^) [27] for 60 min at 37 °C as a kinetic index of ˙OH formation (Figure 6A). To perform the experiment, *p*-NDA (6.25 µM), sodium ascorbate (1 mM), and H_2_O_2_ (1 mM) were diluted in 10 mM lithium cacodylate buffer (pH 7.4) with 100 mM KCl, and Cu(II)-NDI complex, **HP2Cu**, or **PE2Cu** (5 µM), was added as the last reagent. Both the Cu(II)-NDI ligands can produce hydroxyl radical under oxidative conditions with comparable initial rates: v_i_ = (−8.5 ± 0.2) × 10^−8^ M^−1^min^−1^ for **PE2Cu**, and v_i_ = (−6.8 ± 0.2) × 10^−8^ M^−1^min^−1^ for **HP2Cu**.

To prove the generation of superoxide anion O_2_ˉ˙, we performed Nitro Blue Tetrazolium (NBT) assay. NBT is a yellow, water-soluble nitro-substituted aromatic tetrazolium salt that selectively reacts with intracellular superoxide giving rise to blue formazan, which can be easily detected monitoring the increase of absorbance at 560 nm [29]. The experiment was performed by diluting NBT (62.5 µM) in 10 mM lithium cacodylate buffer at pH 7.4 in presence of KCl (100 mM), H_2_O_2_ (1 mM), and ascorbate (1 mM). Cu(II)-NDI complex, **HP2Cu**, or **PE2Cu** (5 µM), was added as the last reactant, and the absorbance increase was monitored continuously for 30 min (Figure 6B). For the first 12 min, the initial rate is significantly equal (v_i_ = (3.1 ± 0.1) × 10^−4^ min^−1^). Subsequently, a steeper increase can be seen for **HP2Cu** (from 12 to 25 min, v_2_ = (5.3 ± 0.2) × 10^−4^ min^−1^), unlike **PE2Cu**, whose rate of NBT reduction decreases (v_2_ = (2.0 ± 0.1) × 10^−4^ min^−1^).

Based on these results, we can conclude that both **HP2Cu** and **PE2Cu** generate hydroxyl radical and superoxide anion, which actively contribute to NA cleavage and/or oxidation.

## 3. Material and Methods

### 3.1. General Information

Solvents and chemicals were purchased from TCI (Tokio, Japan) or Merck (Milano, Italy) and were used as supplied without further purification. DNA and RNA sequences (Table S1) were purchased from Eurogentec (Seraing, Belgium) and dissolved in MilliQ water to prepare 0.5 mM stock solutions. The exact concentrations were then determined by absorption measurements at 260 nm, using the calculated epsilon. The solutions were stored at −20 °C.

HPLC analyses (λ = 256 nm) were performed using an Agilent system SERIES 1260 (Agilent Technologies, Santa Clara, CA, USA) with a Waters XSelect^®^ CSH C18 (2.5 μm, 150 × 4.6 mm) column and a 1.4 mL/min flow rate.

An Agilent Technologies 1260 Infinity preparative HPLC (Agilent Technologies, Santa Clara, CA, USA) provided with a diode array UV-vis detector was used for compounds’ purification. The preparative column was a Waters XSelect^®^ CSH Prep C18 OBDTM (5 μm, 100 × 30 mm) which worked at 30 mL/min. For all analyses and purifications, we employed 0.1% trifluoroacetic acid in water and acetonitrile as solvents. For the analytical analysis, the following method A was used: isocratic gradient over 2 min 95% of H_2_O + 0.1% TFA (5% CH_3_CN), gradually to 40% aqueous solvent over 6 min, then isocratic flow for 4 min. Preparative HPLC was performed by using the methods B and C. B: 95% aqueous over 2 min, gradually decreased to 60% over 14 min, subsequently to 40% in 3 min, then isocratic flow for 2 min; C: 95% aqueous, gradually decreased to 80% over 18 min, then to 70% in other two min.

^1^H- and ^13^C-NMR spectra were recorded on a Bruker Avance 300 MHz (BRUKER, Billerica, MA, USA). UPLC-MS data were recorded using a surveyor UPLC system (Thermo Finnigan, San Jose, CA, USA) equipped with a BEH Acquity UPLC column (1.7 µm) 2.1 × 50 mm, and an LCQ ADV MAX ion-trap mass spectrometer, with an ESI ion source.

UV-visible spectra were measured with an Agilent Cary 60 spectrophotometer (Agilent Technologies, Santa Clara, CA, USA) equipped with an Agilent Technologies Cary Single Cell Peltier temperature controller. The absorbance was recorded from 200 to 900 nm wavelength interval at a scan rate of 200 nm/min and a slit width of 1.5 nm.

Circular Dichroism (CD) analyses were run on a JASCO J-1500 spectropolarimeter (JASCO Corporation, Easton, MD, USA), equipped with a Peltier temperature controller in a quartz cell with a 5 mm path length.

### 3.2. Synthetic Procedures

#### 3.2.1. Synthesis of **HP2**

Compound **1** was synthesized according to a previously published protocol [18]. The imidation of di-bromo substituted naphthalene diimide was performed by dissolving 50 mg of the substrate (0.57 mmol, 1 eq.) into 4 mL of 45:55 mixture of CH_3_COOH:EtOH. Then, 114 µL of 2-(2-aminoethoxy)ethanol (1.14 mmol, 2 eq.) were added and the solution was heated to 120 °C for 5 min with the assistance of the microwaves (Scheme 2, step a). The reaction mixture was cooled down to room temperature and the solvent was removed under reduced pressure. Crude was dissolved in distilled water and extracted three times with dichloromethane (3 × 50 mL). The organic phase was dried over sodium sulfate, filtered, and the solvent removed under vacuum. Compounds **2** and **3** were obtained as a mixture that was used for the subsequent step without further purification.

In a round-bottomed flask, 400 mg of the solid mixture were dissolved into 10 mL of dimethylacetamide, and 550 µL of diethylentriamine (5.3 mmol, 8 eq.) were added (Scheme 2, step b). The reaction mixture was heated to 45 °C for three h, then the solvent was removed under vacuum. Crude was purified through preparative HPLC, using method B, described in the previous section. All the HPLC fractions of purified **HP2** were collected, the solvent was removed under vacuum, then **HP2** was resuspended in acidic water (HCl 10% *v*/*v*) and dried again under vacuum obtaining an orange solid (yield 55%). **HP2** HPLC t_R_ = 5.48 min (96.6%). **HP2** UPLC-ESI-MS (positive mode): 544.33 (**HP2** + H)^+^, 566.33 (**HP2** + Na)^+^. **HP2·HCl** ^1^H-NMR (300 MHz, D_2_O) δ ppm: 7.91 (1H, d, *J* = 7.85 Hz), 7.71–7.69 (2H, m), 4.16–4.12 (4H, m), 3.95–3.91 (2H, m), 3.77–3.68 (4H, m), 3.62–3.59 (8H, m), 3.47–3.44 (4H, m), 3.38–3.33 (2H, m). ^13^C-NMR (75 MHz, D_2_O) δ ppm: 165.0, 163.2, 162.9, 162.8, 151.3, 130.7, 127.7, 126.7, 124.6, 124.5, 121.8, 118.8, 118.0, 99.7, 71.6, 71.5, 67.2, 60.3, 60.3, 54.2, 46.7, 44.5, 39.8, 39.2, 38.8, 35.3.

#### 3.2.2. Synthesis of **PE2**

In a round-bottomed flask, 257 mg of compound **4** (0.45 mmol, 1 eq.), synthesized as previously reported [19], were suspended in 70 mL of acetonitrile and 113 µL of 2-(2-aminoethoxy)ethanol (1.14 mmol, 2.5 eq.) were added. The mixture was heated to 80 °C and refluxed for four h (Scheme 3, step a). Subsequently, the solvent was removed under vacuum and the resulting red solid was resuspended in a saturated solution of hydrogen carbonate extracting the product three times with dichloromethane (3 × 300 mL). Compounds **5** and **6** were obtained as a mixture and were used for the subsequent step without further purification.

In a round-bottomed flask, 300 mg of the solid mixture were dissolved into 10 mL of dimethylformamide, and 484 µL of diethylentriamine (4.7 mmol, 8 eq.) were added. The reaction mixture was heated to 45 °C and stirred overnight (Scheme 3, step b). The solvent was removed under vacuum and the crude, containing compound **5** and **PE2**, was purified through reverse phase column chromatography, following method C described in the previous section. All the HPLC fractions of purified **PE2** were collected, the solvent was removed under vacuum, then **PE2** was resuspended in acidic water (HCl 10% *v*/*v*) and dried again under vacuum obtaining a violet solid (yield 33%). **PE2** HPLC t_R_ = 4.77 min (92.3%). **PE2** UPLC-ESI-MS (positive mode): 307.17 (**PE2** + 2H)^2+^, 613.50 (**PE2** + H)^+^. **PE2·3HCl** ^1^H-NMR (300 MHz, D_2_O) δ ppm: 7.87 (1H, s), 7.81 (1H, s), 4.52–4.36 (4H, m), 3.97–3.90 (2H, m), 3.90–3.82 (2H, m), 3.69–3.68 (6H, m), 3.45–3.40 (8H, m), 3.36–3.34 (2H, m) 2.96 (12H, s). ^13^C-NMR (75 MHz, D_2_O) δ ppm: 165.6, 165.5, 163.7, 163.7, 149.3, 148.0, 124.8, 124.7, 120.9, 120.5, 119,1, 117.0, 114.2, 102.6, 71.7, 68.9, 60.4, 55.4, 55.3, 46.7, 44.4, 43.2, 42.0, 38.6, 35.3, 35.2, 35.1. **5** HPLC t_R_ = 5.16 min (92%). **5** UPLC-ESI-MS (positive mode): 256.67 (**5** + 2H)^2+^, 512.33 (**5** + H)^+^. **5·2HCl** ^1^H-NMR (300 MHz, D2O) δ ppm: 8.16 (1H, d, *J* = 8.0 Hz), 7.93 (1H, d, *J* = 8.0 Hz), 7.82 (1H, s), 4.43–4.36 (4H, m), 3.85–3.81 (4H, m), 3.70–3.63 (4H, m), 3.45–3.37 (4H, m), 2.92 (12H, s). ^13^C-NMR (75.4 MHz, D2O) δ ppm: 165.1, 163.7, 163.5, 163.1, 152.0, 130.9, 128.5, 126.5, 124.9, 124.2, 121.9, 120.6, 118.4, 98.3, 71.8, 68.6, 60.4, 55.3, 54.8, 43.2, 43.2, 42.3, 35.6, 35.1.

#### 3.2.3. Synthesis of **HP2Cu** and **PE2Cu** Complexes

Both the complexes were obtained following the method previously reported [13]. In total, 56 mg of **HP2** (0.096 mmol), or 30 mg of **PE2Cu** (0.041 mmol), were dissolved in 10 mL of distilled water. A stoichiometric amount of Cu(ClO_4_)_2_ hexahydrate was added (35.6 mg and 15.4 mg, respectively) and the solution was stirred for 30 min. The pH of the solution was adjusted to 7 and an excess of KPF_6_ was added forcing the complex precipitation. The mixture was hypercentrifugated to remove the water, then the solid was washed three times with cooled diethyl ether and dried under vacuum.

**HP2Cu**, violet solid (yield 63%), ESI-MS (positive mode, direct injection in MeOH): 605.25 (**HP2Cu**)^+^ Cu(II)-specific isotopic signature.

**PE2Cu**, blue solid (yield 82%), ESI-MS (positive mode, direct injection in MeOH): 675.27 (**PE2Cu**)^+^ Cu(II)-specific isotopic signature.

### 3.3. UV–Vis Spectrophotometric Titrations

Spectrophotometric titration experiments were performed in thermostated cells at 25 °C.

To evaluate the formation of the Cu(II)-complexes, spectrophotometric titrations of **HP2** and **PE2** were performed in a quartz cell of 10 cm path length. To a solution of **HP2** or **PE2** NDI-ligands (5 µM, 27 mL) in 0.1 M HEPES buffer (pH 7.4), increasing concentrations of a stock solution of Cu(OTf)_2_ were added (from 0 to 3 equivalents). All the titrations were repeated in triplicate.

To process the data, the molar absorptivity values (corrected for dilution) of the maximum wavelength of free-NDIs and Cu(II)-complexed-NDIs were reported in the function of copper equivalents. The points were interpolated by two straight lines, identifying the point of intersection mathematically (please see Figure S5 to interpolation lines equation).

To identify the binding constant values (Logβ), a competition titration with TREN (10 mM stock solution in distilled water) was performed in a quartz cuvette of 1 cm path length.

To a solution of **HP2Cu** or **PE2Cu** (50 µM, 3 mL) in 0.1 M HEPES buffer at pH 7.4, increasing amounts of tris(2-aminoethyl)amine (TREN, from 0 to 3 equivalents) were added. All the titrations were performed in duplicates. Binding constants were extrapolated processing the titration data with Hyperquad package [30]. For the Cu(II)-TREN complex a Logβ value of 18.8 was considered [20]. A range of wavelengths centered around the maximum of the two CT bands was used and the region of the isosbestic points was excluded. In particular, the ranges 500–540 nm and 550–580 nm were considered for **HP2Cu**, while 580–635 nm and 650–720 nm were used for **PE2Cu**.

### 3.4. Circular-Dichroism (CD) Analysis

CD spectra were recorded on a Jasco J-1500 spectropolarimeter (JASCO Corporation, Easton, MD, USA), equipped with a Peltier temperature controller. A quartz cell of 5 mm optical length was used for data between 230 and 340 nm, with a 1 nm sampling interval. The reported spectra represent the average of three scans, recorded with a scanning speed of 50 nm/min with a response time of 4 s and a bandwidth of 1 nm. The direct CD measurements (θ, in millidegrees) were converted to mean residue molar ellipticity.

The two model-oligonucleotides were folded into G4 structures dissolving 100 µM oligonucleotides in lithium cacodylate buffer (10 mM, pH 7.4) in the presence of different KCl concentrations. The solutions were heated at 95 °C for 5 min, then slowly left cooling down to room temperature for 4 h. Melting experiments were performed after 16 h of incubation at 20 °C of Cu(II)-complexes, **HP2Cu**, **PE2Cu**, or **NDI-Cu-DETA** (10 µM) with G4-folded oligonucleotide (5 µM), in presence of lithium cacodylate buffer (10 Mm, pH 7.4) and KCl (0.1, 1, 10 or 100 mM). The single wavelength (264 nm for TERRA and 290 nm from hTel22) was recorded every 0.5 °C, while a complete spectrum was registered every 2 min increasing the temperature from 20 °C to 90 °C with a gradient of 0.5 °C/min. Tm values were calculated according to the van’t Hoff equation, applied for a two-state transition from a folded to unfolded state, assuming that the heat capacity of the folded and unfolded states are equal [31].

Evaluation of ROS-mediated NA cleavage was performed by CD analysis on copper-complexes **HP2Cu** and **PE2Cu**. Ligands (5 µM), in presence of KCl (100 mM), and lithium cacodylate buffer (10 mM, pH 7.2) were incubated with oligonucleotide (5 µM) and the solution was stored at 20 °C for 16 h.

The starting CD spectra were recorded, then solutions of sodium ascorbate (1 mM) and H_2_O_2_ (1 mM) were added under stirring and a second CD spectrum was recorded after two h of oxidative reaction.

### 3.5. Denaturing PAGE Assay

5′-FAM-TERRA oligonucleotide (Table S1) was suspended in TRIS-HCl 10 mM, pH 7.4, in presence of 100 mM KCl, heat-denatured at 95 °C for 5 min and folded in ice for 30 min. Then, 2 equivalents of Cu(II)-NDI complexes **HP2Cu** or **PE2Cu** (10 µM) were added to folded RNA-G4 (5 µM) and the mixtures were left to equilibrate for 16 h at 20 °C. Next, the solutions were incubated at 37 °C for 2.5 min, 5 min, 15 min, 60 min, or 120 min after the addition of sodium ascorbate 1 mM and H_2_O_2_ 1 mM. The reaction was quenched freezing the solutions in liquid nitrogen after the addition of the denaturing gel loading buffer, containing 95% formamide, 20 mM ethylenediaminetetraacetic acid, and bromophenol blue. All the samples were diluted 1:5 and loaded on a 7 M urea 12% polyacrylamide gel. The reaction products were acquired with Typhoon Trio (GE Healthcare, Chicago, IL, USA).

### 3.6. Characterization of Reactive Oxygen Species Involved

#### 3.6.1. Bleaching of *p*-Nitroso-*N*,*N*-dimethylaniline (*p*-NDA)

The irreversible bleaching of *p*-NDA has been evaluated through spectrophotometric analysis, by monitoring the absorption decrease at 440 nm (ε_440nm_ = 34,200 M^−1^cm^−1^) for 60 min at 37 °C as a kinetic index of hydroxyl radical formation. Sodium ascorbate (1 mM) and H_2_O_2_ (1 mM) were added to a solution of *p*-NDA (6.25 µM) in 10 mM lithium cacodylate buffer (pH 7.4) with 100 mM KCl. The kinetic process was recorded after the addition of Cu(II)-NDI complexes **HP2Cu** and **PE2Cu** (5 µM). The kinetic profiles were corrected for the *p*-NDA bleaching due to sodium ascorbate alone. No effects on *p*-NDA absorbance were highlighted for simple auto-oxidation or by H_2_O_2_ contribution alone, as reported before (data not shown) [27].

The initial rate corresponds to the angular coefficient of the straight line that interpolates the experimental points (*p*-NDA concentrations in the function of time) from 0 to 10 min.

#### 3.6.2. Nitro Blue Tetrazolium (NBT) Assay

Nitro Blue Tetrazolium (NBT) assay was performed to evaluate the generation of O_2_ˉ˙ superoxide anion. The increase of blue formazan concentration was followed by the absorbance rise at 560 nm. Sodium ascorbate (1 mM) and H_2_O_2_ (1 mM) were added to a solution of NBT (62.5 µM) in 10 mM lithium cacodylate buffer (pH 7.4) with 100 mM KCl. The kinetic process was recorded after the addition of Cu(II)-NDI complexes **HP2Cu** and **PE2Cu** (5 µM).

The initial rate (v_i_) corresponds to the angular coefficient of the straight line that interpolates the experimental points (absorbance of blue formazan in the function of time) from 0 to 12 min, while v_2_ has been calculated considering the data from 12 to 25 min.

## 4. Conclusions

Here, we have proposed the design and synthesis of two novel Cu(II)-NDI complexes for the selective cleavage of parallel G4s triggered by ROS. Both **HP2Cu** and **PE2Cu** have shown an extraordinary ability in copper coordination with impressive binding constants at physiological pH, equal to 16.30 ± 0.08 and 17.95 ± 0.02 Log units for **HPCu2** and **PE2Cu**, respectively. These high binding constants ensure the stability of the complex under physiological conditions, making feasible future evaluations in cell-based systems.

As a proof of concept of our aim, we decided to study functional targeting of **HPCu2** and **PE2Cu** on two telomeric G4, hTel22 and TERRA, which differ in G4 topology. They are composed of the same nucleic acid sequence but being a DNA and an RNA-G4, respectively, the different sugars in the backbone (deoxyribose vs ribose) and Uraciles instead Thymines induce a G4 folding into a hybrid topology for hTel22 and a parallel one for TERRA.

The presence of 2-(2-aminoethoxy)ethanol side chains on **HPCu2** and **PE2Cu** complexes cause a selective stabilization of parallel TERRA-G4, compared to hTel22. Moreover, the analysis of the behavior of **PE2Cu** towards hTel22, suggests that the introduction of this Peg-like chain on the naphthalene core, instead of at the imide position, induces a reorganization of the bases and a change of preferential topology of the G4, from hybrid to parallel. The topological shift in favor of parallel is remarkable as it does not unfold under the experimental conditions used.

Preliminary studies on oxidative reactivity on TERRA-G4, followed by CD, suggested that under oxidative conditions (generated in situ by H_2_O_2_ and sodium ascorbate), **HPCu2** and **PE2Cu** catalyze an oxidative pathway. The characteristic dichroic spectrum of parallel G4 is partially retained after 2 h of reaction under oxidative stress conditions, suggesting a localizing NA damage, not solely on guanine sites, which are the nucleobases most susceptible to oxidation.

A preliminary analysis on TERRA oxidative cleavage was performed by denaturing PAGE, highlighting specific cleaving sites induced by **HPCu2** and **PE2Cu** redox catalysis in oxidative stress conditions.

Simple assays have been exploited to identify the ROS potentially involved in the NA cleavage. In particular, the bleaching of *p*-NDA suggests the contribution of hydroxyl radical, while the formation of blue formazan from NBT reduction also indicates the generation of superoxide anion.

These promising results lay a solid mainstay for the further development of selective functional NDI-ligands towards the parallel topology, the only possible one for RNA-G4s, starting to answer to the notable need to exploit these targets for future innovative therapeutic approaches.

## Figures and Tables

**Table 1:** CD analysis of **HP2Cu** and **PE2Cu** binding towards G4 sequences. ΔT_m_ values were measured by CD melting of 5 μM oligonucleotides, in the presence of 10 μM compounds, in 10 mM lithium cacodylate buffer (pH 7.4), at the indicated potassium concentrations.

		Alone	HP2Cu		PE2Cu		NDI-Cu-DETA	
	λ (nm)	T_m_ (°C)	T_m_ (°C)	ΔT_m_ (°C)	T_m_ (°C)	ΔT_m_ (°C)	T_m_ (°C)	ΔT_m_ (°C)
TERRA (K^+^ = 100 mM)	264	81.5 ± 0.7	>90	>8	>90	>8.5	>90	>8.5
TERRA (K^+^ = 10 mM)	264	72.6 ± 0.3	84.2 ± 0.1	+11.6	>90	>17.4	82.6 ± 0.8	+10.0
TERRA (K^+^ = 1 mM)	264	63.7 ± 0.2	74.5 ± 0.4	+10.8	>90	>26.3	79.1 ± 0.5	+15.4
TERRA (K^+^ = 0.1 mM)	264	51.2 ± 0.2	58.5± 0.1	+7.3	75.4 ± 0.2	+24.2	66.4 ± 0.3	+15.2
hTel22 (K^+^ = 100 mM)	290	78.24 ± 0.06	79.2 ± 0.6	+1.0	61.3 ± 0.1	−16.9	82.4 ± 0.2	+4.16
	264	80.3 ± 0.9	83.6 ± 0.4	+3.3	>90	>11.6	89.0 ± 0.2	+8.7
hTel22 (K^+^ = 10 mM)	290	62.52 ± 0.05	66.13 ± 0.07	+3.61	49.2 ± 0.1	−13.3	70.34 ± 0.09	+7.82
	264	66.4 ± 0.3	80 ± 1	+13.6	88.5 ± 0.3	+22.9	84.9 ± 0.3	+18.5

## Data Availability

The authors confirm that the data supporting the findings of this study are available within the article and its Supplementary Materials.

## Supplementary Materials

The following are available online, Table S1. Nucleic acid sequences used in the present studies. Figure S1. Competition titration with TREN. Table S2. CD analysis of hTel22 after its folding in the presence of Cu(II)-NDI ligands. Figure S2. CD spectra of hTel22 in presence of **NDI-Cu-DETA**. Figure S3. CD spectra of hTel22 in presence of **HP2Cu**. Figure S4. Denaturing PAGE assay. Figure S5. Molar absorption profiles of NDI-ligand/Cu(II) titration. Additional data, i.e., HPLC purity data, ESI-MS spectra, and ^1^H- and ^13^C-NMR characterization for the here synthesized NDIs, are reported on pages S6–S13.

## Sample Availability

Samples of the compounds are available from the authors.
