# Supplementary material for: Selective Binding and Redox-Activity on Parallel G-Quadruplexes by Pegylated Naphthalene Diimide-Copper Complexes
Source: Molecules. 2021 Aug 19;26(16):5025. doi: 10.3390/molecules26165025 (PMC8397950; doi:10.3390/molecules26165025)

Supplementary Material

Selective Binding and Redox-Activity on Parallel G-Quadruplexes by Pegylated Naphthalene Diimide-Copper Complexes

Valentina Pirota [1,2,*], Enrico Lunghi [1], Alessandra Benassi [1,2], Emmanuele Crespan [3], Mauro Freccero [1] and Filippo Doria [1,*]

1 Department of Chemistry, University of Pavia, via Taramelli 10, 27100 Pavia, Italy; enrico.lunghi01@universitadipavia.it (E.L.); alessandra.benassi01@universitadipavia.it (A.B.); mauro.freccero@unipv.it (M.F.)
2 "G-Quadruplexes as INnovative ThERApeutiC Targets" (G4-INTERACT), Universal Scientific Education and Research Network (USERN), via Taramelli 10, 27100 Pavia, Italy
3 Institute of Molecular Genetics IGM-CNR "Luigi Luca Cavalli-Sforza", via Abbiategrasso 207, 27100 Pavia, Italy; crespan@igm.cnr.it
* Correspondence: valentina.pirota@unipv.it (V.P.); filippo.doria@unipv.it (F.D.)

Table of Contents

	Page
Oligonucleotide Sequences (Table S1)	S2
Competition titration with TREN (Figure S1)	S2
CD analysis of hTel22 after its folding in the presence of Cu(II)-NDI ligands (Table S2)	S2
CD spectra of hTel22 in presence of **NDI-Cu-DETA** (Figure S2) or **HP2Cu** (Figure S3)	S3
Denaturing PAGE assay (Figure S4)	S4
Molar absorption profiles of NDI-ligand/Cu(II) titration (Figure S5)	S5
HPLC Purity Analysis	S6
ESI-MS Data	S7-S10
^{1}H-NMR and ^{13}C-NMR Characterization	S11-S13

Table S1. Nucleic acid sequences used in the present studies.

Name	Nature	ε at 260 nm ($cm^{-1}M^{-1}$)	Sequences (5’-3’)	Length
**TERRA**	RNA	237500	AGGGUUAGGGUUAGGGUUAGGG	22 nucleotides
**hTel22**	DNA	228500	AGGGTTAGGGTTAGGGTTAGGG	22 nucleotides
**5’-FAM-TERRA**	RNA	237500	*FAM*-AGGGUUAGGGUUAGGGUUAGGG	22 nucleotides

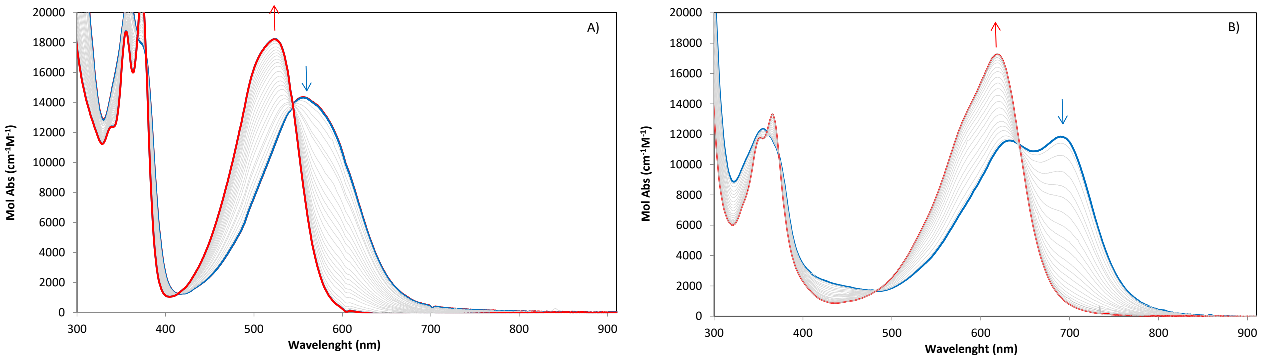

Figure S1. Uv-vis spectra taken upon titration of A) **HP2Cu** or B) **PE2Cu** (50 µM) with increasing equivalents of TREN (from 0 to 3 eq.) in 0.1 M HEPES buffer at pH 7.4, 25 °C.

Table S2. CD-melting experiment performed after the folding of hTel22 already in the presence of Cu(II)-NDIs. ΔTm values were measured by CD melting of 5 µM oligonucleotides, in the presence of 10 µM compounds, in 10 mM lithium cacodylate buffer (pH 7.4) with 100 mM or 10 mM KCl.

		Alone	HP2Cu		PE2Cu		NDI-Cu-DETA	
	λ (nm)	T_m (°C)	T_m (°C)	ΔT_m (°C)	T_m (°C)	ΔT_m (°C)	T_m (°C)	ΔT_m (°C)
**hTel22 (K^+ = 100 mM)**	290	78.24 ± 0.06	79.3 ± 0.1	+1.1	45 ± 2	- 33	85.6 ± 0.1	+7.4
	264	80.3 ± 0.9	84.5 ± 0.3	+4.2	> 90°C	> 11.64	>90°C	> 11.64
**hTel22 (K^+ = 10 mM)**	290	62.52 ± 0.05	67.0 ± 0.5	+4.5	36.4 ± 0.1	- 26.1	73.2 ± 0.1	+10.7
	264	66.4 ± 0.3	78.8 ± 0.2	+12.4	> 90°C	> 23.6	84.7 ± 0.5	+18.3

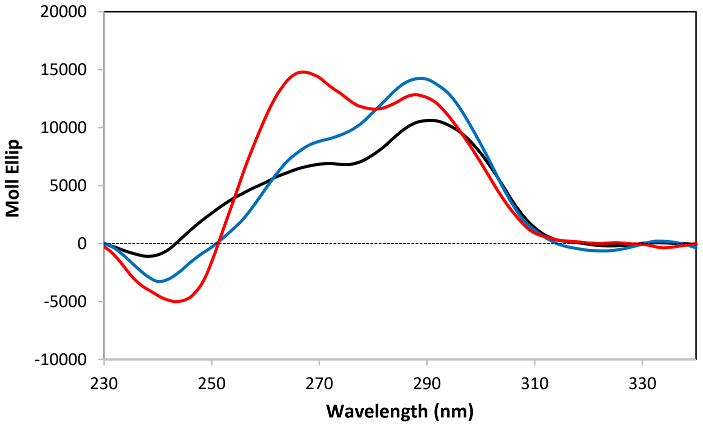

Figure S2. CD spectra of 5 μM hTel22 alone (black line), after the addition of 2 eq. of **NDI-Cu-DETA**, left to equilibrate at room temperature for 16 h (blue line) and after the hTel22 G4 folding already in the presence of 2 eq. of **NDI-Cu-DETA** (red line) in 10 mM lithium cacodylate buffer at pH 7.4 with 100 mM KCl.

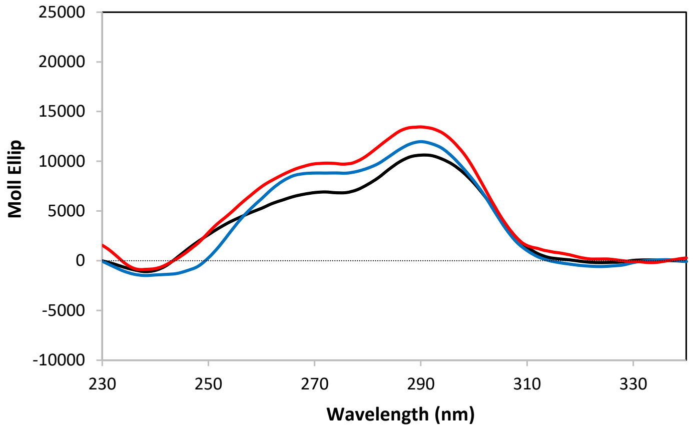

Figure S3. CD spectra of 5 μM hTel22 alone (black line), after the addition of 2 eq. of **HP2Cu**, left to equilibrate at room temperature for 16 h (blue line) and after the hTel22 G4 folding already in the presence of 2 eq. of **HP2Cu** (red line) in 10 mM lithium cacodylate buffer at pH 7.4 with 100 mM KCl.

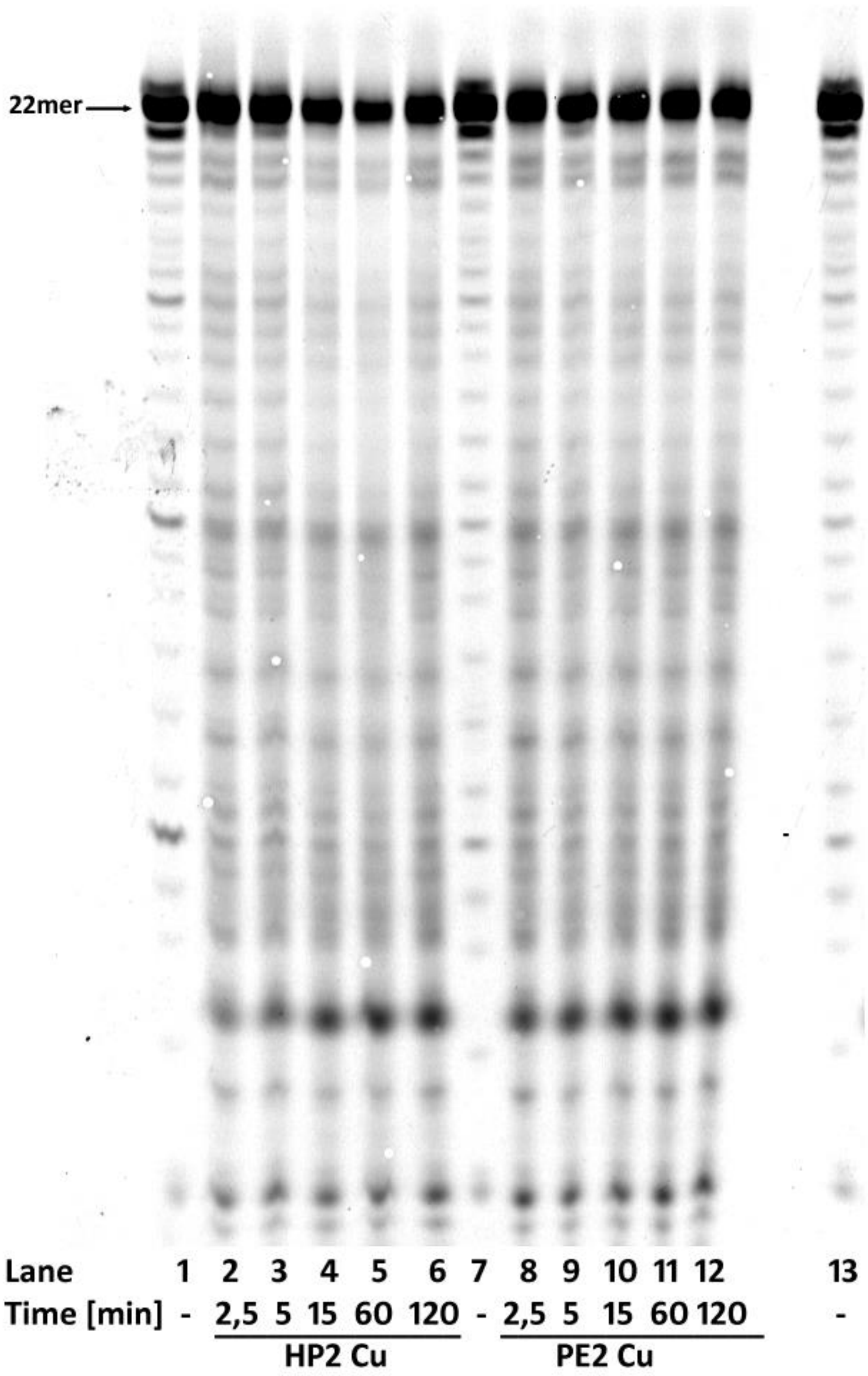

Figure S4. Denaturing polyacrylamide gel electrophoresis showing the oxidative cleavage of TERRA RNA-G4 (5 µM) by Cu(II)-NDI ligands (10 µM) in 10 mM TRIS-HCl buffer (pH 7.4), with 100 mM KCl.
Lanes 1-7: **HP2Cu** + TERRA only (lane 1) or in the presence of sodium ascorbate (1 mM) and H_2O_2 (1 mM) at different reaction times (lanes 2-6). Lanes 8-12: **PE2Cu** + TERRA only (lane 7) or in the presence of sodium ascorbate (1 mM) and H_2O_2 (1 mM) at different reaction times (lanes 9-12). Lane 13: TERRA alone.

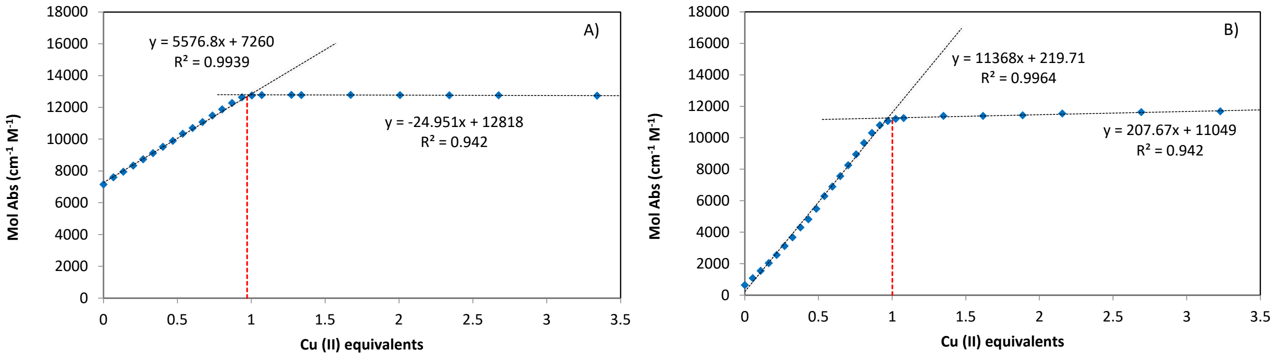

Figure S5. Molar absorption profiles at A) 555 nm for **HP2Cu** complexation and at B) 690 nm for **PE2Cu** complexation. On the graph are reported the equations of the interpolation lines used to derive the point of intersection.

HPLC purity analysis

For HPLC analysis and characterization, the following method was used: flow 1.4 mL/min, isocratic gradient over 2 min 95% of H_2O + 0.1% TFA (5% CH_3CN), gradually to 40% aqueous solvent over 6 min, then isocratic flow for 4 min (λ = 256 nm).

HP2 (t_R = 5.48 min; 96.6 %)

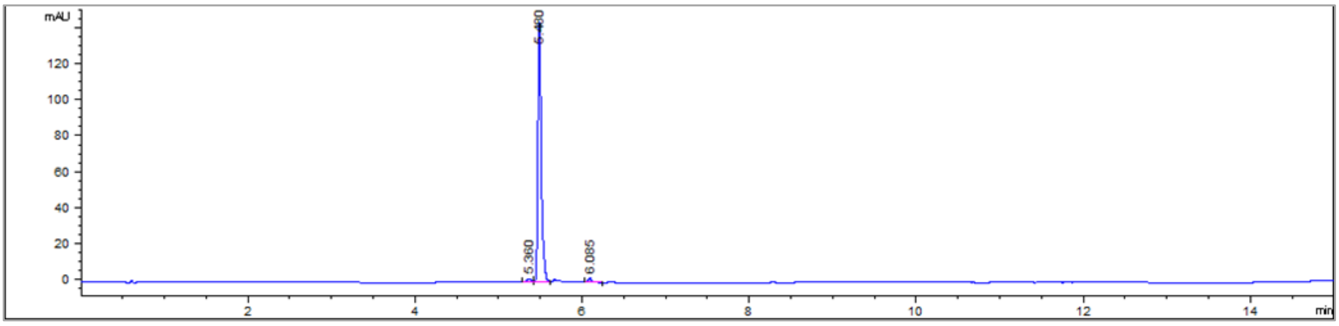

PE2 (t_R = 4.77 min; 92.3 %)

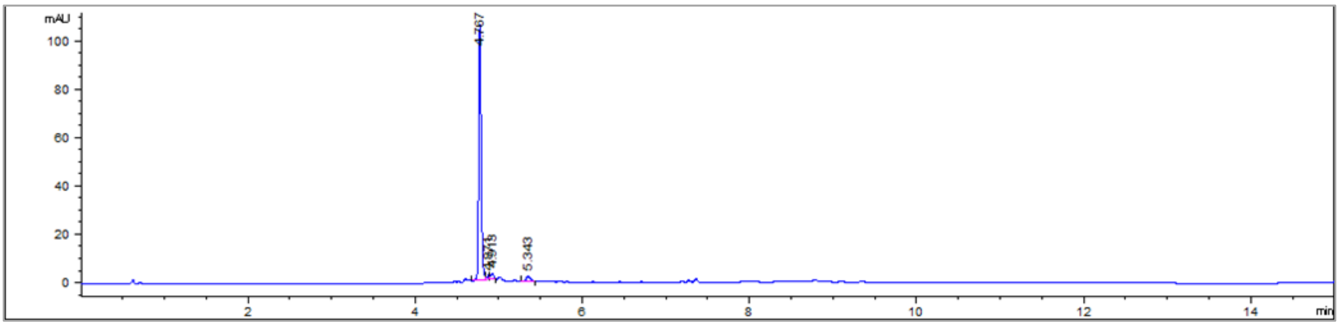

Compound 5 (t_R = 5.16 min; 92.4 %)

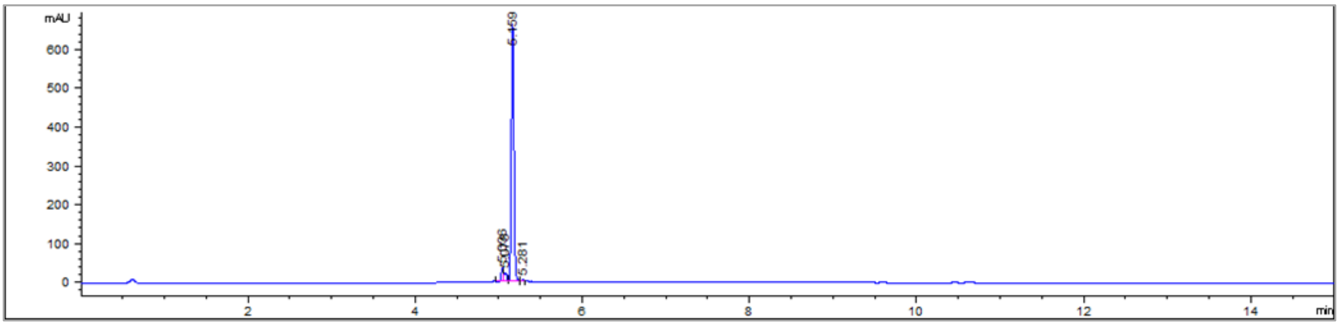

ESI-MS Data

HP2 (Mol. Wt.: 543.58 u.m.a.)

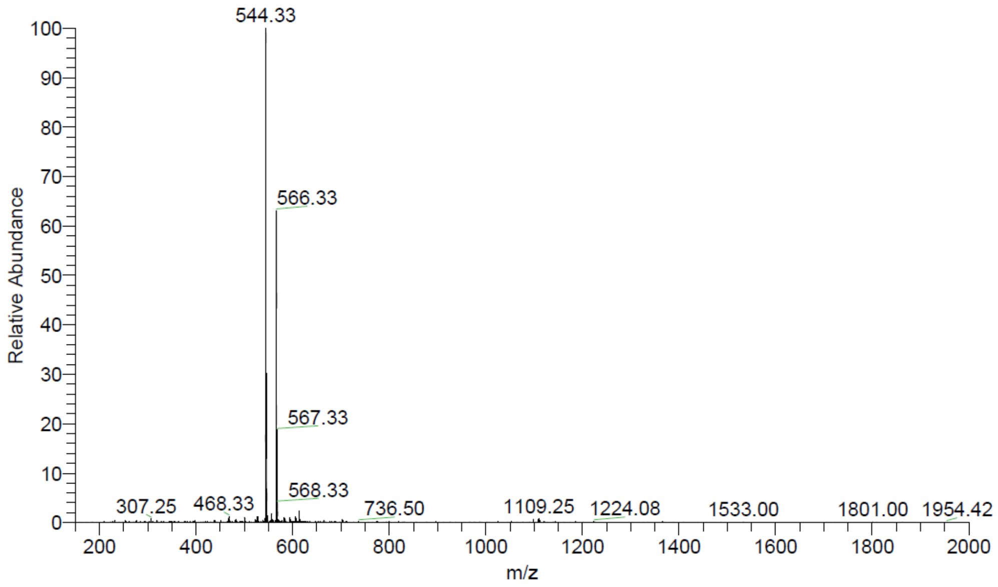

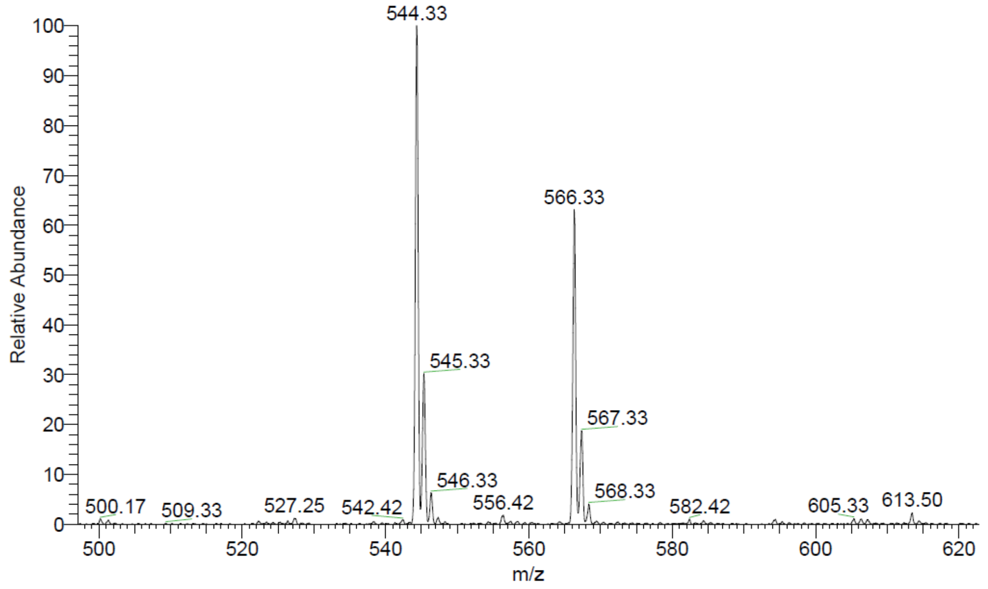

HP2Cu (Mol. Wt.:606.11)

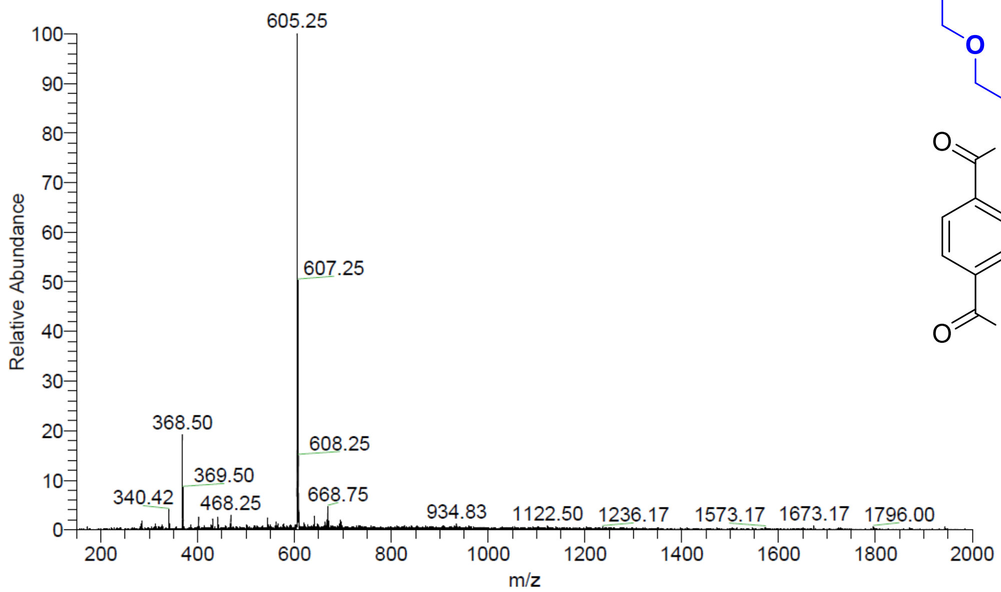

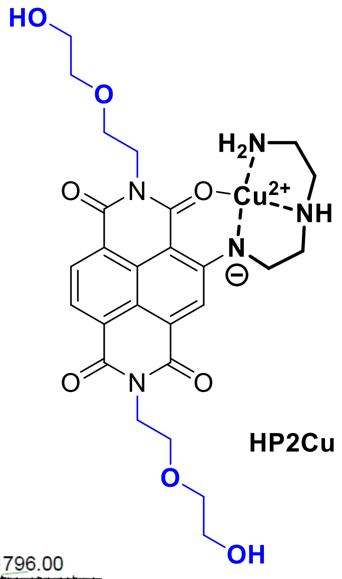

HP2Cu

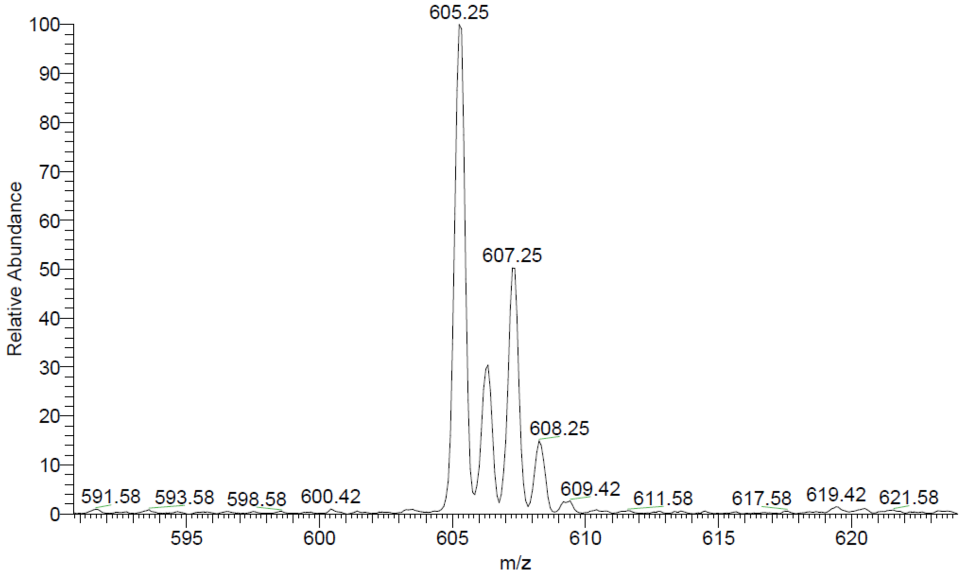

PE2 (Mol. Wt.: 612.73 u.m.a.)

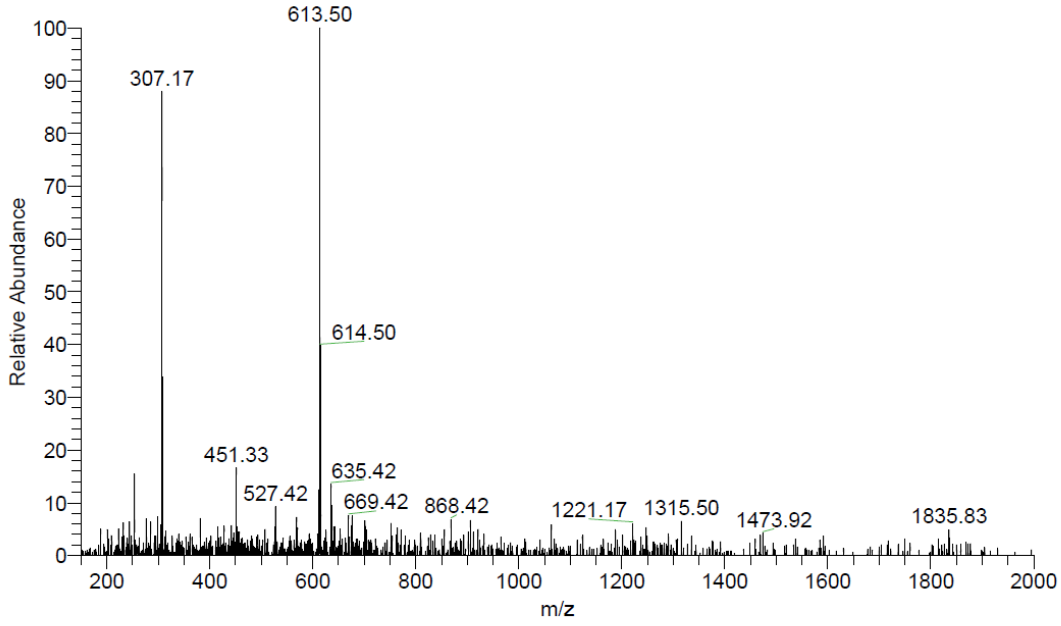

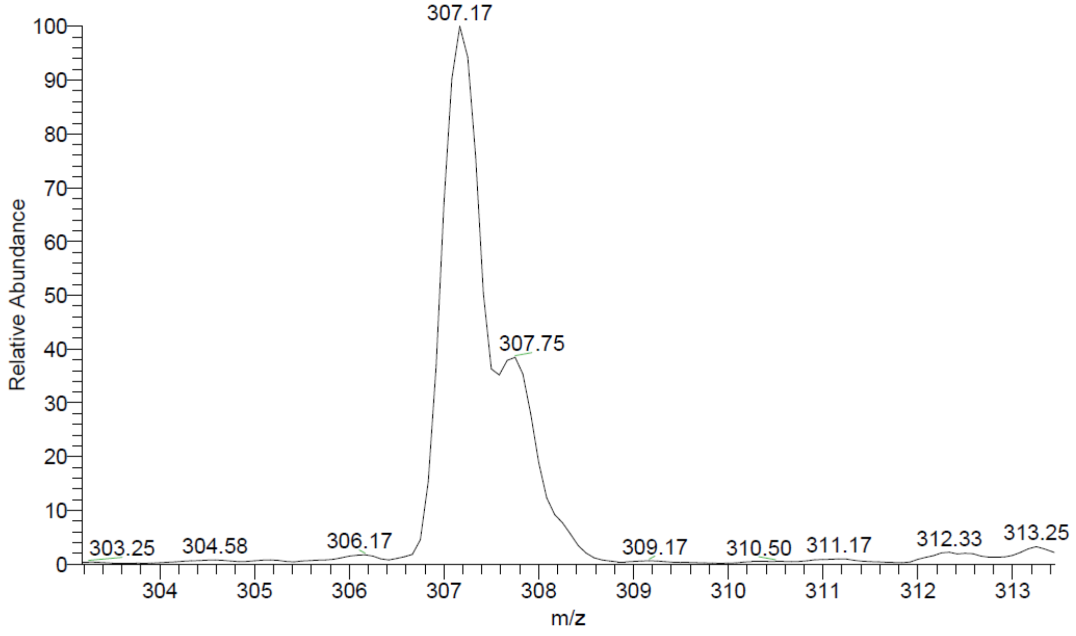

PE2Cu (Mol. Wt.: 675.27 u.m.a.)

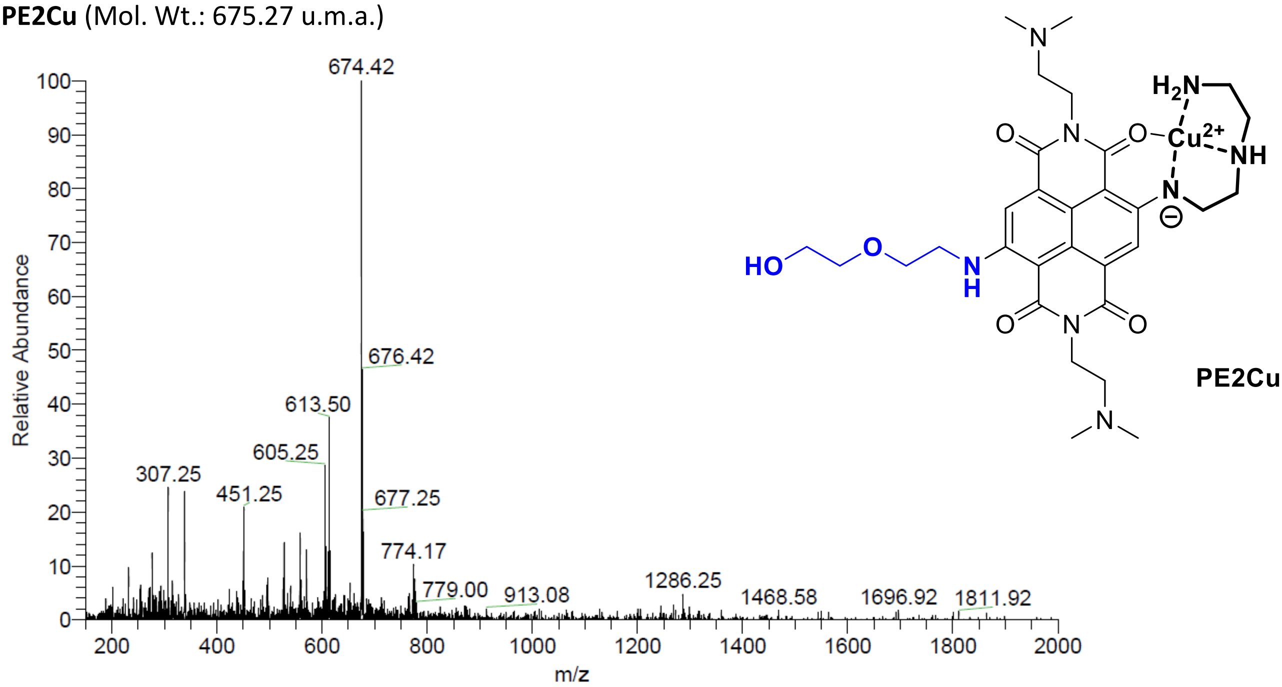

PE2Cu

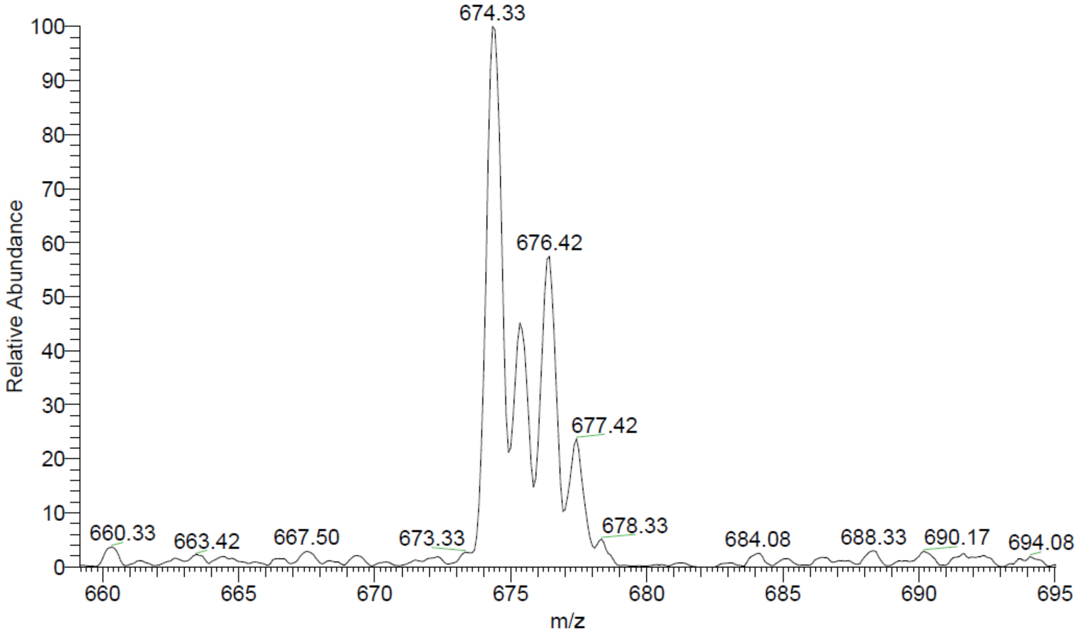

Compound 5 (Mol. Wt.: 511.58 u.m.a.)

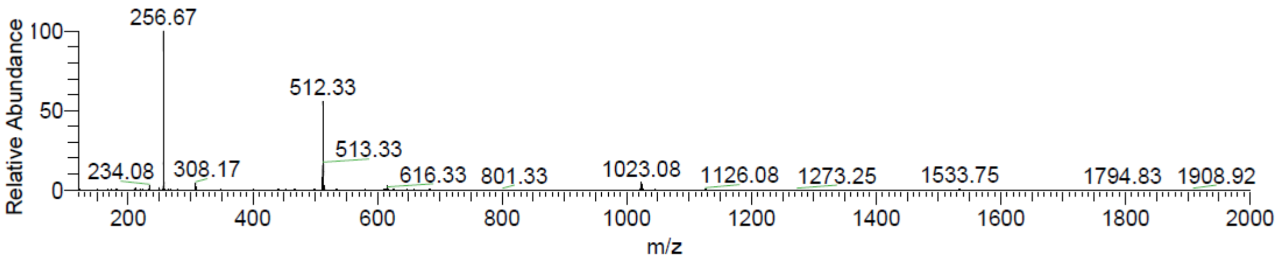

^{1}H-NMR and ^{13}C-NMR Characterization

HP2. ^{1}H-NMR (300 MHz, D_2O)

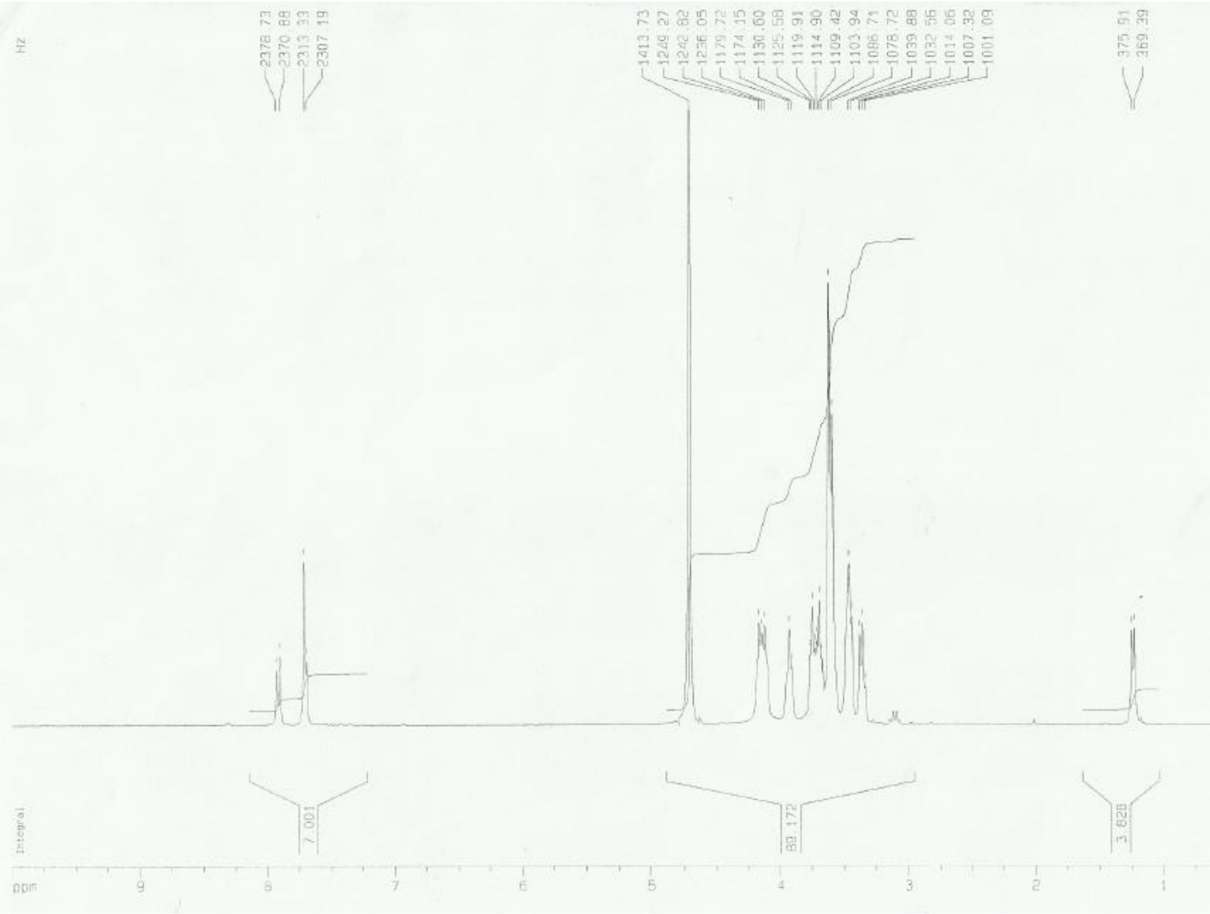

HP2. ^{13}C-NMR (75 MHz, D_2O)

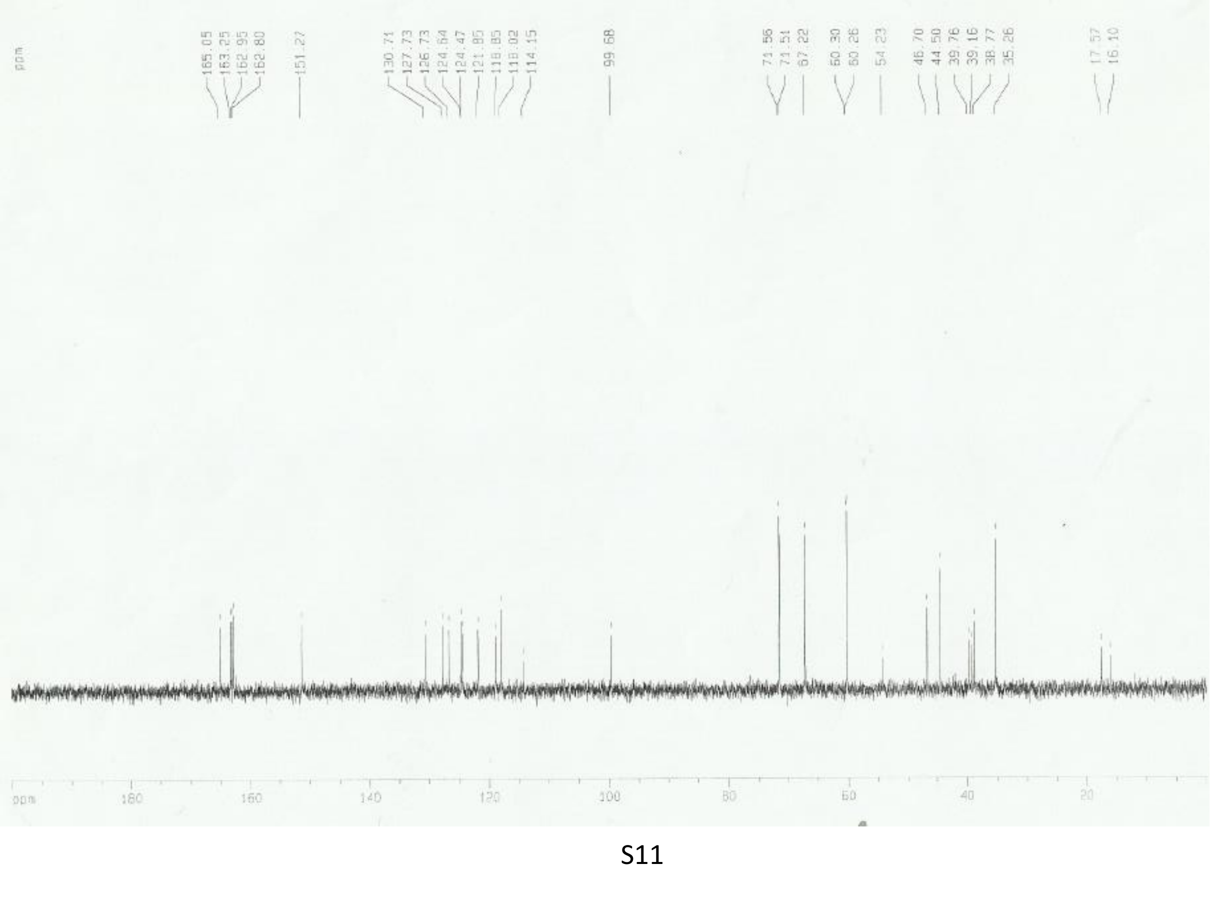

PE2. ^{1}H-NMR (300 MHz, D_2O)

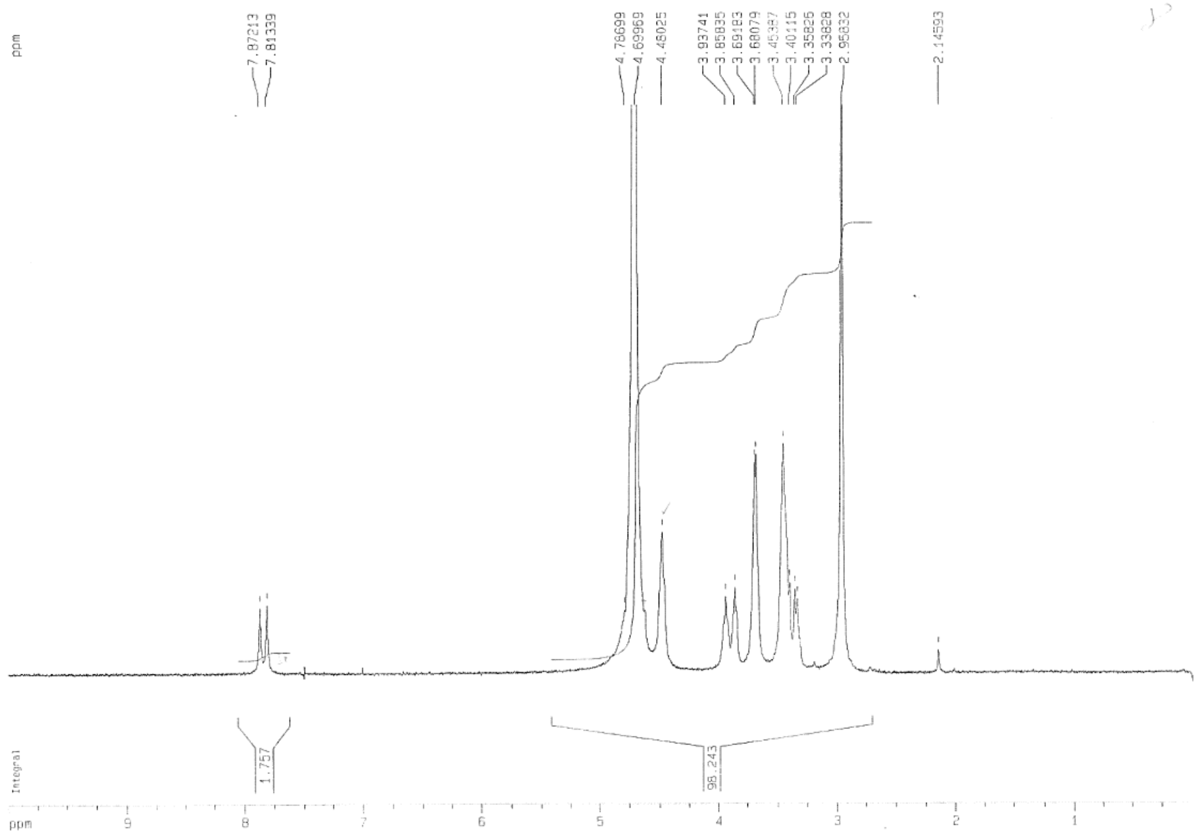

PE2. ^{13}C-NMR (75 MHz, D_2O)

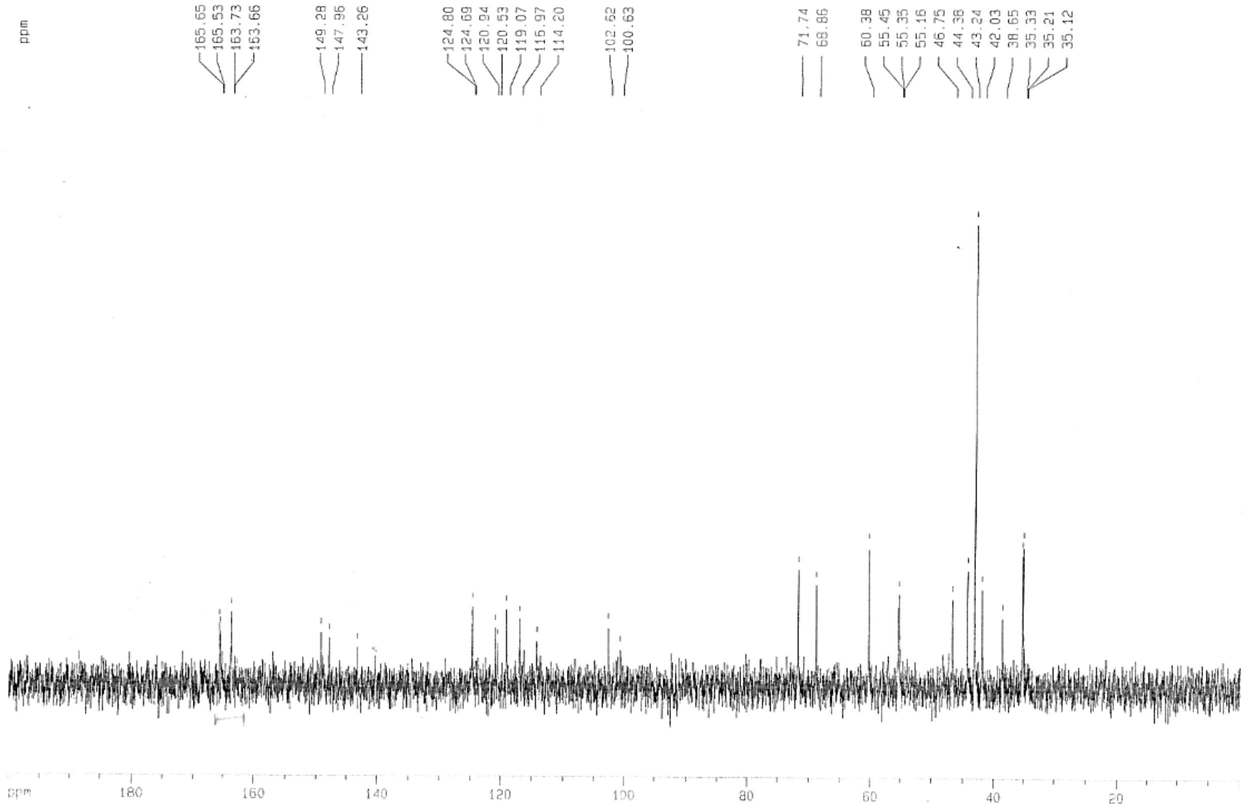

Compound 5. ^{1}H-NMR (300 MHz, D_2O)

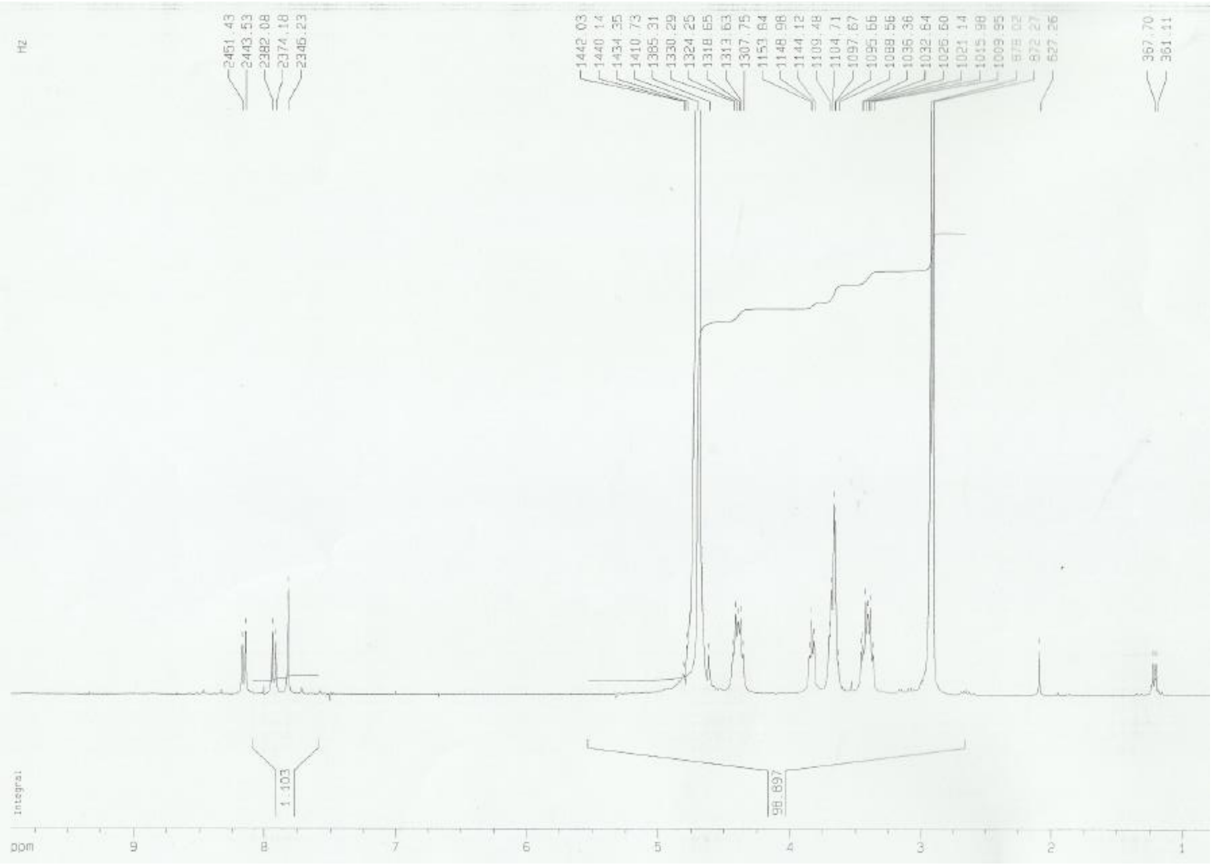

Compound 5. ^{13}C-NMR (75 MHz, D_2O)

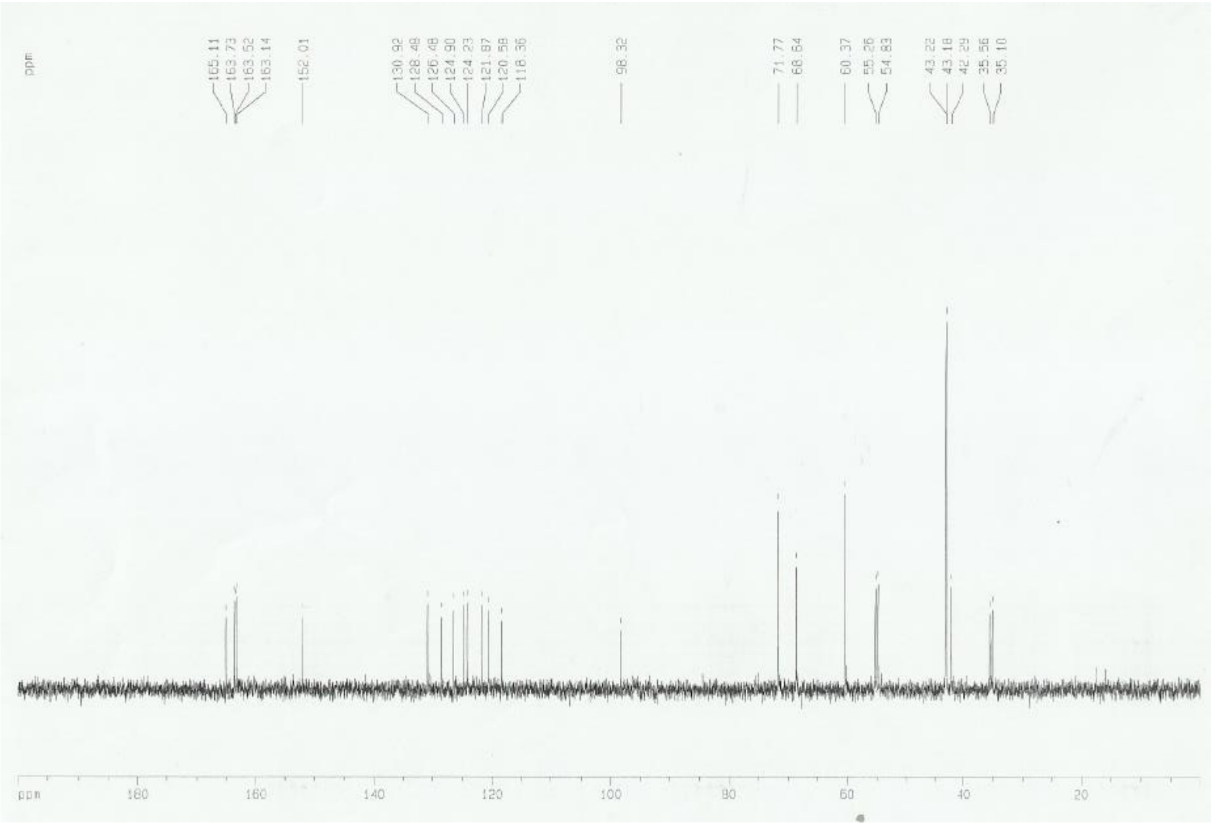

Supplement: Supplementary file 1 [file molecules-26-05025-s001.zip › molecules-1291831-supplementary.pdf]
